# Phase-matching-free parametric oscillators based on two-dimensional semiconductors

**DOI:** 10.1038/s41377-018-0011-3

**Published:** 2018-05-18

**Authors:** Alessandro Ciattoni, Andrea Marini, Carlo Rizza, Claudio Conti

**Affiliations:** 1 Consiglio Nazionale Delle Ricerche (CNR-SPIN), Via Vetoio 10, 67100 L’Aquila, Italy; 2 Department of Physical and Chemical Sciences, University of L’Aquila, Via Vetoio 10, 67100 L’Aquila, Italy; 3 Institute for Complex Systems (ISC-CNR), Via dei Taurini 19, 00185 Rome, Italy; 4 ICFO-Institut de Ciencies Fotoniques, The Barcelona Institute of Science and Technology, 08860 Castelldefels, Barcelona Spain; 5 Department of Industrial and Information Engineering and Economics, University of L’Aquila, Via G. Gronchi 18, I-67100 L’Aquila, Italy; 6 Department of Physics, University Sapienza, Piazzale Aldo Moro 5, 00185 Rome, Italy

## Abstract

Optical parametric oscillators are widely used as pulsed and continuous-wave tunable sources for innumerable applications, such as quantum technologies, imaging, and biophysics. A key drawback is material dispersion, which imposes a phase-matching condition that generally entails a complex design and setup, thus hindering tunability and miniaturization. Here we show that the burden of phase-matching is surprisingly absent in parametric micro-resonators utilizing mono-layer transition-metal dichalcogenides as quadratic nonlinear materials. By the exact solution of nonlinear Maxwell equations and first-principle calculations of the semiconductor nonlinear response, we devise a novel kind of phase-matching-free miniaturized parametric oscillator operating at conventional pump intensities. We find that different two-dimensional semiconductors yield degenerate and non-degenerate emission at various spectral regions due to doubly resonant mode excitation, which can be tuned by varying the incidence angle of the external pump laser. In addition, we show that high-frequency electrical modulation can be achieved by doping via electrical gating, which can be used to efficiently shift the threshold for parametric oscillation. Our results pave the way for the realization of novel ultra-fast tunable micron-sized sources of entangled photons—a key device underpinning any quantum protocol. Highly miniaturized optical parametric oscillators may also be employed in lab-on-chip technologies for biophysics, detection of environmental pollution and security.

## Introduction

Optical nonlinearity in photonic materials enables a large number of applications such as frequency conversion^1,2^, all-optical signal processing^3,4^, and non-classical sources^5,6^. Parametric down-conversion (PDC) furnishes tunable sources of coherent radiation^7–14^ and generators of entangled photons and squeezed states of light^15,16^. In traditional configurations, a nonlinear crystal with broken centrosymmetry and second-order nonlinearity is used to sustain PDC;^7–12^ more recently, effective PDC was reported in centrosymmetric crystals with third-order nonlinearity^13,14^ and semiconductor micro-cavities^17–19^.

**Fig. 1:**
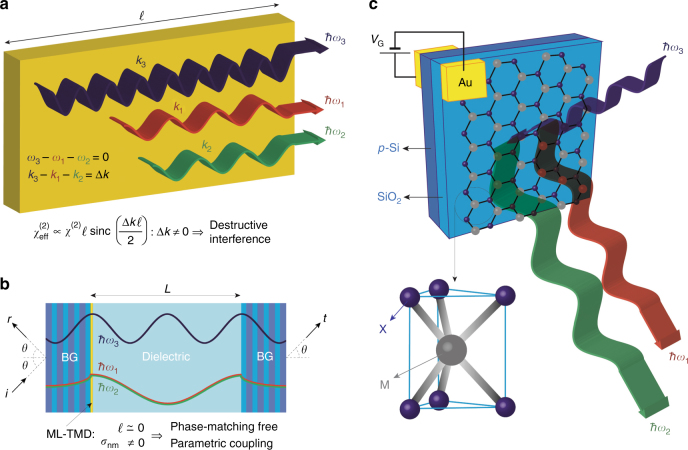
Phase-matching-free micron-sized parametric oscillators. **a** Schematic illustration for conventional three-wave parametric coupling in bulk nonlinear crystals. The effective quadratic susceptibility $\chi _{\mathrm {eff}}^{\left( 2 \right)}$ is heavily affected by the mismatch Δ*k* among the wavevectors *k*_*m*_ = *n*_*m*_*ω*_*m*_/c of the pump (3), signal (1), and idler (2) waves, whose destructive interferenceΔ*k* ≠ 0 hinders parametric coupling. **b** Sketch of the ML-TMD-based parametric oscillator. The cavity is assembled using two Bragg mirrors separated by a dielectric layer, and the ML-TMD is placed onto the left mirror. The incident (i) pump field produces both reflected (r) and transmitted (t) pump, signal and idler fields by means of the ML-TMD quadratic surface conductivity *σ*_*nm*_ ≠ 0. The mutual dephasing $\Delta \phi = \Delta {\it{k}}\ell$ among these three waves becomes negligible within the atomic thickness of the nonlinear ML-TMD (Δϕ ≈ 10^−2^, see Supplementary Material) because $\ell \ll \lambda$, thus enabling phase-matching-free (i.e., free from the momentum conservation requirement Δ*k* = 0) parametric coupling. **c** Sketch of the geometry of MX_2_ ML-TMDs. Fast modulation is enabled by extrinsic doping by a gate voltage, with gold contacts applied between the ML-TMD and the Bragg mirror.

Since three-wave parametric coupling is intrinsically weak, one can only achieve low oscillation thresholds by using doubly or triply resonant optical cavities. In addition, parametric effects are severely hampered by the destructive interference among the three waves propagating with different wavenumbers *k*_1,2,3_ in the dispersive nonlinear medium because of a generally non-vanishing wavevector mismatch Δ*k* = *k*_3_ − *k*_2_ −*k* _1_ (see Fig. 1a). To avoid this highly detrimental effect, the use of phase-matching (PM) strategies is imperative, i.e., following the standard nonlinear optics terminology, fulfillment of momentum conservation Δ*k* = 0 to prevent destructive interference. The commonly adopted birefringence-PM method^20^ is critically sensitive to the nonlinear medium orientation. Quasi-PM^21,22^ exploits the momentum due to a manufactured long-scale periodic reversal of the sign of the nonlinear susceptibility, which cannot be easily applied in miniaturized systems. In semiconductors, PM is achieved by S-shaped energy-momentum polariton dispersion in the strong coupling regime for excitons and photons^23,24^ that is only accessible at low temperatures and large pump angles. Cavity PM^25^, also denoted “relaxed” PM^26^, occurs in Fabry–Perot micro-cavities with cavity length $\ell$ shorter than the coherence length *π*/Δ*k*; this technique can be used to drastically reduce the effective quadratic susceptibility $\chi _{\mathrm {eff}}^{\left( 2 \right)}$ (see Fig. 1a). All of the above-mentioned PM techniques require a non-trivial experimental design and setup that is further constrained by the need for resonant operation.

In this manuscript, we show that two-dimensional (2D) materials with high quadratic nonlinearity, currently emerging as important nonlinear photonic elements^27–29^, open up unprecedented possibilities for tunable parametric micro-sources. Remarkably, when illuminated by different visible and infrared waves, such novel 2D materials provide negligible dispersive dephasing Δϕ owing to their atomic-scale thickness $\ell \ll \lambda$, where *λ* is the optical wavelength (see Fig. 1b). In turn, the three waves interacting within the 2D material do not undergo destructive interference due to the surface-like nonlinear interaction. Hence, the PM requirement in the standard nonlinear optics jargon (i.e., the momentum conservation requirement Δ*k* = 0), is removed here. Furthermore, such “phase-matching-free” devices turn out to be very versatile and compact, with additional tunability afforded by electrical gating of 2D materials, which provides ultrafast electrical-modulation functionality.

The most famous 2D material, graphene, is not the best candidate for PDC owing to the centrosymmetric structure. In principle, a static external field can be used to break centrosymmetry and induce a $\chi _{\mathrm {eff}}^{\left( 2 \right)}$, but the spectrally flat absorption of graphene remains severely detrimental for PDC. Recent years have witnessed the rise of transition metal dichalcogenides (TMDs) as promising photonic 2D materials. TMDs possess several unusual optical properties dependent on the number of layers. Bulk TMDs are semiconductors with an indirect bandgap, but the optical properties of their monolayer (ML) counterpart are characterized by a direct bandgap ranging from ~1.55 to ~1.9 eV^30–32^ that is beneficial for several opto-electronic applications^33^. In addition, ML-TMDs have broken centrosymmetry and can thus be used to facilitate second-order nonlinear processes^34–38^. Here, we study PDC in micro-cavities embedded with ML-TMDs; we find that the cavity design is extremely flexible compared to standard parametric oscillators due to phase-matching-free operation (see Fig. 1a, b). We demonstrate that at conventional infrared pump intensities, parametric oscillation occurs in wavelength-sized micro-cavities incorporating ML-TMDs. We show that the mode selectivity of doubly resonant cavities enables one to engineer the output signal and idler frequencies; these frequencies are tuned by the pump incidence angle and can be modulated electrically by an external gate voltage.

## Materials and methods

### Parametric down-conversion for MX_2_

We calculate the linear and PDC mixing surface conductivities of MX_2_ starting from the tight-binding (TB) Hamiltonian for the electronic band structure^39^. Since the properties of infrared photons with energies smaller than the bandgap are determined by small electron momenta around the K and K′ valleys, we approximate the full TB Hamiltonian as a sum of *k* ⋅ *p* Hamiltonians of first and second order *H*_0_(*k*,*τ*,*s*), where *k* is the electron wavenumber and *τ* and *s* are the valley and spin indices, respectively. We then derive the light-driven electron dynamics through a minimal coupling prescription leading to the time-dependent Hamiltonian $H_0\left[ {{\mathbf{k}} + \left( {{\it{e}}{\mathrm{/}}\hbar } \right){\mathbf{A}}\left( {\it{t}} \right),\tau ,{\it{s}}} \right]$, where *e* is the electron charge, *ħ* is the reduced Planck constant, and *A*(*t*) is the radiation potential vector, which is used to obtain Bloch equations for the interband coherence and the population inversion. Finally, by solving perturbatively the Bloch equations for ML-TMDs in the weak excitation limit, we obtain the surface current density **K**(*t*) after integration over reciprocal space,

1 $$\begin{array}{ccccc}\\ {\mathbf{K}}\left( {\it{t}} \right) = & {\Re} \left\{ {\mathop {\sum}\limits_{{\it{j}} = 1}^3 {\left[ {\hat \sigma ^L\left( {\omega _{\it{j}}} \right){\mathbf{E}}_{\it{j}}e^{ - i\omega _{\it{j}}{\it{t}}}} \right]} + \hat \sigma ^{\left( {1,2} \right)}{\mathbf{E}}_1{\mathbf{E}}_2e^{ - i\omega _3{\it{t}}}} \right.\\ \\ &\left. { + \hat \sigma ^{\left( {1,3} \right)}{\mathbf{E}}_1^ \ast {\mathbf{E}}_3e^{ - i\omega _2{\it{t}}} + \hat \sigma ^{\left( {2,3} \right)}{\mathbf{E}}_2^ \ast {\mathbf{E}}_3e^{ - i\omega _1{\it{t}}}} \right\}\\ \end{array}$$

where $\hat \sigma ^L\left( {\omega _{\it{j}}} \right)$ (*j* = 1, 2, 3) and $\hat \sigma ^{\left( {{\it{l,m}}} \right)}$ (*l, m* = 1, 2, 3) are the linear and PDC surface conductivity tensors, respectively. Note that our approach is based on the independent-electron approximation and is thus fully justified only for infrared photons far from exciton resonances occurring at photon energies higher than 1.5 eV^40,41^.

### Parametric oscillations

The signal, idler, and pump fields, labeled with subscripts 1, 2, and 3, respectively, have frequencies *ω*_*n*_ satisfying *ω*_1_ + *ω*_2_ = *ω*_3_. By the transfer matrix approach, a full electromagnetic analysis of the cavity (see Supplementary Material) yields the equations

2 $$\begin{array}{l}\Delta _1{\it{Q}}_1 + \tilde \sigma _{23}{\it{Q}}_2^ \ast {\it{Q}}_3 = 0,\\ \Delta _2{\it{Q}}_2 + \tilde \sigma _{13}{\it{Q}}_1^ \ast {\it{Q}}_3 = 0,\\ \Delta _3{\it{Q}}_3 + \tilde \sigma _{12}{\it{Q}}_1{\it{Q}}_2 = {\it{P}}_3\end{array}$$

where *Q*_1_,* Q*_2_, *Q*_3_ are complex amplitudes proportional to the output fields produced by the pump field, which is proportional to the amplitude *P*_3_. Here, $\tilde \sigma _{{\it{nm}}}$ are scaled quadratic conductivities for the MX_2_ ML-TMD and

3 $$\Delta _{\it{n}} = \tilde \sigma _{\it{n}} - \frac{{\it{c}}}{{\omega _{\it{n}}}}{\it{q}}_{\it{n}}\left( {\frac{{{\it{r}}_{\it{n}}^{\left( R \right)} - 1}}{{{\it{r}}_{\it{n}}^{\left( R \right)} + 1}} + \frac{{{\it{r}}_{\it{n}}^{\left( R \right)}e^{i{\it{q}}_{\it{n}}{\it{L}}} - e^{ - i{\it{q}}_{\it{n}}{\it{L}}}}}{{{\it{r}}_{\it{n}}^{\left( R \right)}e^{i{\it{q}}_{\it{n}}{\it{L}}} + e^{ - i{\it{q}}_{\it{n}}{\it{L}}}}}} \right)$$

are parameters characterizing the linear cavity, where $\tilde \sigma _{\it{n}}$ are scaled linear surface conductivities, ${\it{q}}_{\it{n}} = \left( {\omega _{\it{n}}{\mathrm{/}}{\it{c}}} \right)\sqrt {\varepsilon \left( {\omega _{\it{n}}} \right) - \sin ^2\theta }$ are the longitudinal wavenumbers inside the dielectric slab, *ε*(*ω*) is the relative permittivity of the dielectric slab, *θ* is the pump incidence angle and ${\it{r}}_{\it{n}}^{\left( R \right)}$ are the complex reflectivities for right illumination of the left Bragg mirror (with vacuum and the dielectric slab on its left and right side). It is worth stressing that the wavevector mismatch Δ*k* = *k*_3_ − *k*_1_ − *k*_2_ does not appear in the basic cavity Eq. (2) because the ML-TMDs are treated as 2D materials with surface-like conductivity. In principle, such media possess an atomic-scale thickness of $\ell = 0.65\;{\mathrm {nm}}$, with the resulting wavevector mismatch Δ*k* producing a finite but negligibly small phase-shift Δϕ ≈ 10^−2^ among the three waves (see Supplementary Material for further details). In turn, such a phase-shift (which does not appear in our formulation based on a surface-like nonlinearity) does not affect parametric coupling (by the destructive interference of the fields), and the phase-matching constraint is heavily relaxed. Parametric oscillations (POs) are solutions of Eqs. (2) with *Q*_1_ ≠ 0 and *Q*_2_ ≠ 0, and in this case, the compatibility of the first two equations yields (see Supplementary Material)

4 $$\left| {{\it{P}}_3} \right|^2 \ge \frac{{\Delta _1\Delta _2^ \ast }}{{\tilde \sigma _{23}\tilde \sigma _{13}^ \ast }}\left| {\Delta _3} \right|^2$$

which is the leading PO condition. As the right hand side of Eq. (4) is generally a complex number, for the realization of PO, we have the condition

5 $$\arg \left( {\frac{{\Delta _1}}{{\tilde \sigma _{23}}}} \right) = \arg \left( {\frac{{\Delta _2}}{{\tilde \sigma _{13}}}} \right)$$

Equation (5) can be physically interpreted as a locking of the phase difference $\arg {\it{Q}}_1 - \arg {\it{Q}}_2^ \ast$ allowing the signal and idler to oscillate. Once Eq. (5) is satisfied, Eq. (4) provides the pump threshold for the onset of PO. Due to the small absolute magnitude of the nonlinear surface conductivities, the cavity parameters |Δ_*n*_| must be minimized to achieve a feasible threshold. This can be obtained by choosing the doubly resonant condition for signal and idler corresponding to the minima of |Δ_1_| and |Δ_2_|, respectively. For these minima to be very small, $\left| {{\it{r}}_1^{\left( R \right)}} \right|$ and $\left| {{\it{r}}_2^{\left( R \right)}} \right|$ are required to be very close to one. Such a constraint can be satisfied by the use of a suitable Bragg mirror design, with the stop-band centered at half of the pump frequency *ω*_3_/2 since, in this case, the signal and idler fields experience a large mirror reflectance.

## Results and discussion

**Fig. 2:**
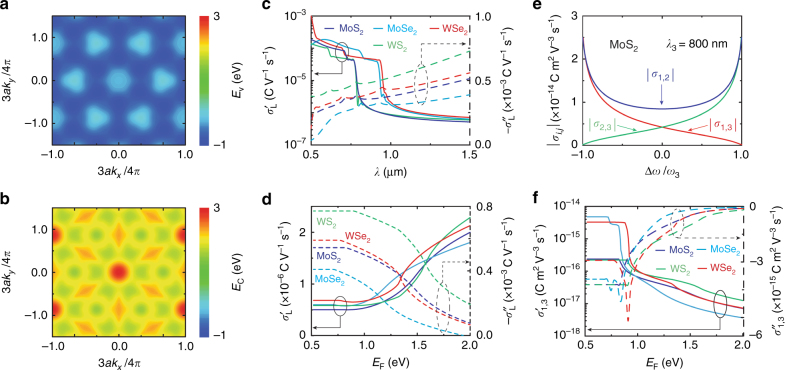
Electronic and optical properties of MX_2_. **a**, **b** Valence *E*_V_(**k**) and conduction *E*_C_(**k**) energy bands for MoS_2_, where *k* is the electron wave-vector and *a* = 3.19 Å is the lattice parameter. **c**, **d** Dependence of the linear surface conductivities of ML-TMDs on **c** the vacuum wavelength *λ* (for intrinsic doping *E*_F_ = 0) and on **d** the Fermi level *E*_F_ ensuing from extrinsic doping (at *λ* = 1.6 μm). **e** PDC mixing surface conductivities of MoS_2_ at *λ*_3_ = 800 nm as a function of the angular frequency mismatch of down-converted signal and idler waves Δ*ω* = *ω*_1_ − *ω*_2_ rescaled to the pump angular frequency *ω*_3_. **f** Dependence of the real and imaginary parts of the PDC mixing conductivity *σ*_1,3_ for MX_2_ ML-TMDs on the Fermi level *E*_F_ for *λ*_1_ = *λ*_2_ = 1.6 μm and *λ*_3_ = 0.8 μm.

The structure of ML-TMDs is formed by two hexagonal lattices of chalcogen atoms embedding a plane of metal atoms arranged at trigonal prismatic sites located between chalcogen neighbors^32^. Figure 1c shows the lattice structure for MX_2_ ML-TMDs (M = Mo, W, and X = S, Se), and Fig. 2a, b show the valence and conduction bands for MoS_2_ as obtained from tight-binding calculations^39^. The electronic band structure of other MX_2_ materials is considered to be qualitatively similar. The direct bandgap is ~1.5 eV, which implies optical transparency for infrared radiation; the linear surface conductivity has a very small real part (corresponding to absorption) and a higher imaginary part at infrared wavelengths. Figure 2c shows the wavelength dependence of the linear surface conductivities of MX_2_. In the presence of an external pump field with angular frequency *ω*_3_, the ML-TMD second-order nonlinear processes lead to the generation of down-converted signal and idler waves with angular frequencies *ω*_1_ and *ω*_2_, such that *ω*_3_ = *ω*_1_ + *ω*_2_. Figure 2e illustrates the PDC mixing surface conductivities for MoS_2_. Both linear and nonlinear conductivities are calculated by a perturbative expansion of the tight-binding Hamiltonian for MX_2_ (see Methods and Supplementary Material). For infrared photons with energy smaller than the bandgap, extrinsic doping by an externally applied gate voltage (see Fig. 1c) modifies the optical properties, leading to increased absorption due to free-carrier collisions and to smaller PDC mixing conductivities. Figure 2d, f show the dependence of the linear and nonlinear surface conductivities on the Fermi level *E*_F_. As detailed below, extrinsic doping generally leads to a decrease in PDC efficiency.

Figure 1b shows the parametric oscillator design incorporating ML-TMDs. The cavity consists of a dielectric slab (thickness *L*) surrounded by two Bragg grating mirrors (BGs); the ML-TMD is placed on the left BG inside the cavity. The cavity is illuminated from the left by an incident (i) pump field (frequency *ω*_3_), and the oscillator produces both reflected (r) and transmitted (t) signal and idler fields with frequencies *ω*_1_ = (*ω*_3_ + Δ*ω*)/2 and *ω*_3_ = (*ω*_3_ − Δ*ω*)/2, where Δ*ω* is the beat-note frequency of the parametric oscillation (PO).

**Fig. 3:**
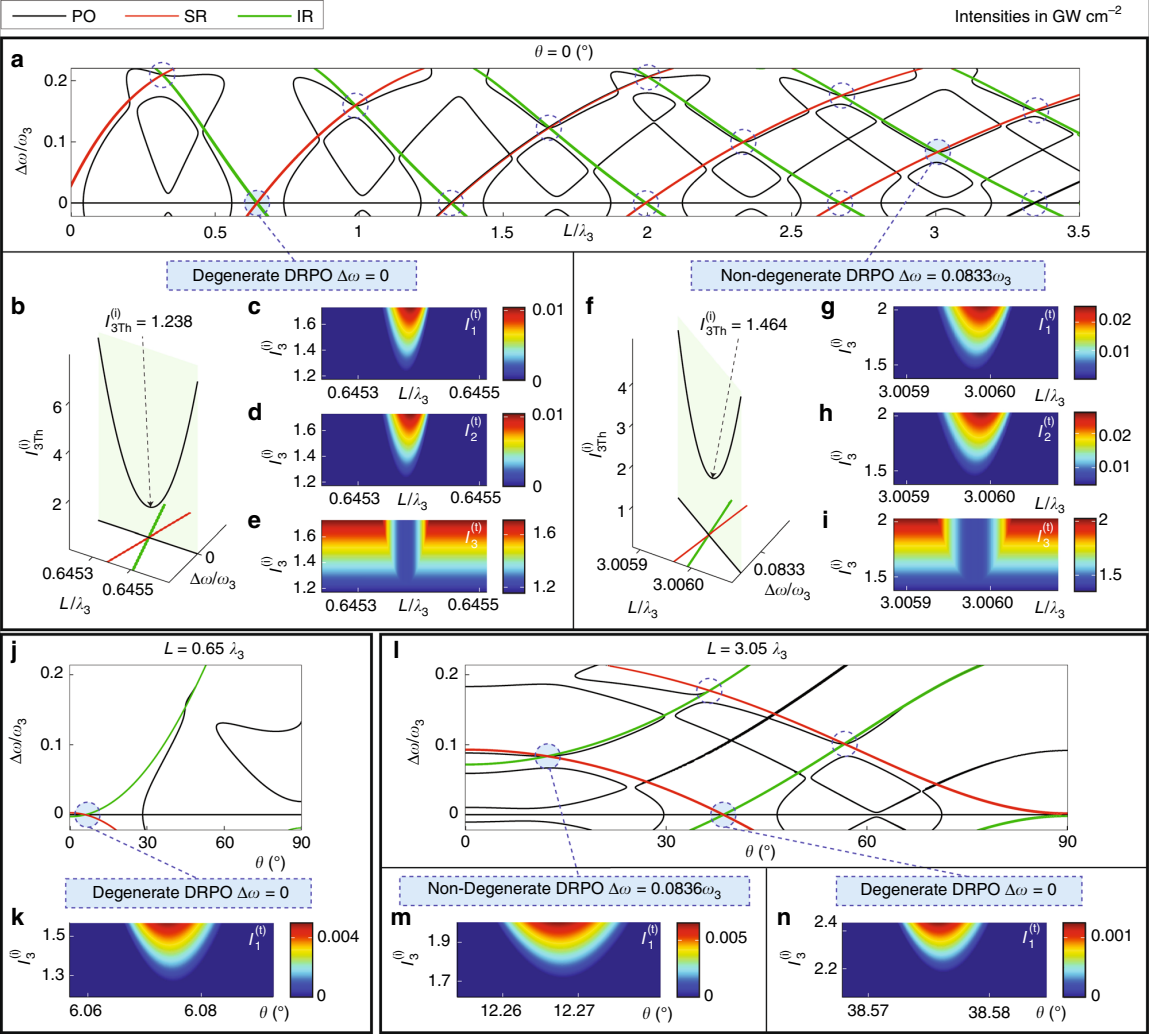
Parametric oscillations. Analysis of the doubly resonant parametric oscillations (DRPOs) of a cavity (with PMMA as the cavity dielectric) illuminated by a *λ*_3_ = 780 nm pump with micron-sized Bragg mirrors whose stop band is centered at 1560 nm. In **a**–**h**, the cavity length is used as a tuning parameter for normal incidence *θ* = 0, whereas in **j**–**n**, the incidence angle *θ* is the tuning parameter for the two assigned cavity lengths. **a** Identification of DRPOs as the intersection between the parametric oscillation (PO) curve and signal resonance (SR) and idler resonance (IR) curves in the (*L*/*λ*_3_, Δ*ω*/*ω*_3_) plane. **b**–**e** Intensity analysis of the degenerate (Δ*ω* = 0) DRPO located at ${\it{L}} \simeq 0.645\;\lambda _3$ comprising the plots of **b** the intensity threshold ${\it{I}}_{3Th}^{\left( i \right)}$ vs. *L*/λ_3_ and Δ*ω*/*ω*_3_ and **c**–**e** the intensities ${\it{I}}_1^{\left( t \right)},{\it{I}}_2^{\left( t \right)},{\it{I}}_3^{\left( t \right)}$ of the transmitted signal, idler, and pump fields as functions of the scaled cavity length *L*/*λ*_3_ and the incident pump intensity ${\it{I}}_1^{\left( i \right)}$. **f**–**i** Intensity analysis of the non-degenerate (Δ*ω* ≠ 0) DRPO located at ${\it{L}} \simeq 3\lambda _3$ (the panels are analogous to those in **b**–**e**). **j** DRPO analysis in the (*θ*, Δ*ω*/*ω*_3_) plane for *L* as in **b**–**e**. Note that the degenerate DRPO occurs at a small angle *θ*. **k** Signal intensity analysis of the DRPO showing a feasible *θ* range. **l** DRPO analysis in the (*θ*, Δ*ω*/*ω*_3_) plane for *L* as in **f**–**i** revealing a variety of DRPOs at different angles *θ*. **m**, **n** Signal intensity analysis for the two DRPOs identified in **l.**

As detailed in the Materials and methods, the cavity equations for the fields do not contain the wavevector mismatch Δ*k*. Indeed, due to their atomic thickness, ML-TMDs are not optically characterized by a refractive index but rather by a surface conductivity. Hence, the parametric coupling produced by the quadratic surface current in ML-TMDs is not hampered by dispersion; thus, no PM condition is required. To observe signal and idler generation, only the PO condition must be satisfied along with the signal resonance (SR) and idler resonance (IR) conditions, leading to a significant reduction in the intensity threshold (see Methods). Since there is no PM requirement, such conditions can be met by adjusting either the cavity length *L* or the pump incidence angle *θ* as tuning parameters. For SR and IR, one needs highly reflective mirrors for both signal and idler (see Materials and methods), as realized by locating the stop band of the micron-sized BGs at half of the pump frequency *ω*_3_/2. Figure 3 shows the PO analysis for a cavity composed of two BGs with polymethyl methacrylate (PMMA) and MoS_2_ deposited on the left mirror. The infrared pump has a wavelength of *λ*_3_ = 780 nm, which lies in the same spectral region showing very pronounced nonlinear properties for MoS_2_ (see Fig. 2e). The BGs are tuned with their stop bands centered at 1560 nm ( = 2*λ*_3_). In Fig. 3a–i, we consider the case of normal incidence *θ* = 0 and plot the PO (black), SR (red), and IR (green) curves in the (*L*/*λ*_3_,Δ*ω*/*ω*_3_) plane. Doubly resonant POs (DRPOs) corresponding to the intersection points of these three curves are labeled by dashed circles. Therefore, for normal incidence of the pump, degenerate (Δ*ω* = 0) and non-degenerate (Δ*ω* ≠ 0) DRPOs exist at specific cavity lengths. Note that such oscillations also occur for sub-wavelength cavity lengths (*L* < *λ*_3_). Each oscillation starts when the incident pump intensity ${\it{I}}_3^{\left( i \right)}$ is increased above a threshold ${\it{I}}_{3Th}^{\left( i \right)}$ (see Materials and methods). Figure 3b–i shows the threshold for two specific degenerate and non-degenerate DRPOs. Figure 3b, f shows the thresholds (black curves on the shadowed vertical planes) corresponding to the PO (black) curves; one can observe that the minimum thresholds occur at SR and IR (identified by the intersection between the red and green curves). The minimum intensity thresholds are on the order of GW cm^−2^, with the non-degenerate DRPO threshold greater than the degenerate DRPO threshold because the reflectivity of the Bragg mirror is maximum at Δ*ω* = 0 (i.e., at half the pump frequency, as discussed above). Figure 3c–e (and, analogously, Fig. 3g–i) shows the basic DRPO features by plotting the intensities ${\it{I}}_1^{\left( t \right)},{\it{I}}_2^{\left( t \right)},{\it{I}}_3^{\left( t \right)}$ of the transmitted signal, idler, and pump fields as functions of the scaled cavity length *L*/*λ*_3_ and the incident pump intensity. Note that, in the considered example, the range of *L*/*λ*_3_ where the oscillation actually occurs is rather narrow due to the high reflectivity of the adopted BG.

We emphasize that tuning of the PO may be realized by adjusting the pump incidence angle *θ*, with negligible effect on the oscillation thresholds. In Fig. 3j–n, we analyze the DRPOs by using *θ* as a tuning parameter for a given cavity length. In particular, in Fig. 3j, k, we consider a cavity with a fixed length, as in Fig. 3b–e. The PO, SR, and IR curves of Fig. 3j intersect at a degenerate DRPO point at $\theta \simeq 6^{\circ}$. In Fig. 3k, we plot the transmitted signal intensity ${\it{I}}_1^{\left( t \right)}$ as a function of the pump incidence angle and intensity ${\it{I}}_3^{\left( i \right)}$; one can observe that the intensity threshold is comparable to the case shown in Fig. 3b–e, with PO occurring for a range of angles *θ* on the order of a hundredth of a degree, which is experimentally feasible. We show similar results in Fig. 3m, n, where the non-degenerate DRPO of Fig. 3f–i is investigated for a cavity with a slightly different length and is shown to exist at a finite incident angle with unchanged note-beat frequency Δ*ω*. A more accurate analysis of Fig. 3l also reveals that, for a given *L*, the cavity sustains multiple DRPOs (both degenerate and non-degenerate) at different incidence angles *θ*. In Fig. 3n, we plot the transmitted intensity of a degenerate DRPO that grows with pump intensity above the ignition threshold.

**Fig. 4:**
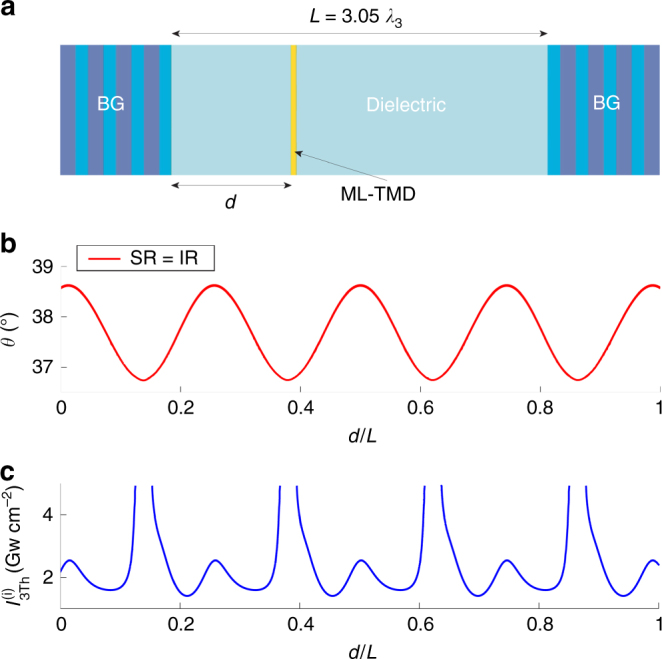
Effect of ML-TMD location on the parametric oscillations. **a** Sketch of the parametric oscillator geometry with the ML-TMD placed at a distance *d* from the right mirror. **b** Incidence angle *θ* of the degenerate (Δ*ω* = 0) DRPOs as a function of the normalized distance *d*/*L*. The degenerate DRPOs are triggered by the same pump as above (*λ*_3_ = 780 *nm*), and they occur at each *d* since, due to the physical coincidence of the signal and idler fields, the SR and IR conditions coincide and the PO condition is automatically satisfied (see Materials and methods). **c** Pump intensity threshold ${\it{I}}_{3Th}^{\left( i \right)}$ of the DRPOs reported in **b** as a function of *d*/*L.*

Until now, our analysis has been based on the basic oscillator geometry sketched in Fig. 1b, where the ML-TMD is placed on top of the right mirror. It is, however, also instructive to investigate the dependence of the PO phenomenology on the location of the ML-TMD inside the cavity. Consequently, we consider a different parametric oscillator design whose geometry is sketched in Fig. 4a, with the same Bragg mirrors and cavity dielectric (of thickness *L* = 3.05*λ*_3_) as above but with the ML-TMD placed at a distance 0 < *d* < *L* from the left mirror. For simplicity, we focus here on degenerate DRPOs (Δ*ω* = 0), triggered by the same pump as above (*λ*_3_ = 780 nm), as in this case, due to the physical coincidence of the signal and idler fields, the SR and IR conditions coincide and the PO condition is automatically satisfied (see Materials and methods). In Fig. 4b, we plot the SR = IR curve identifying the incidence angle *θ* at which the DRPO occurs as a function of the normalized distance *d*/*L*. Note that the PO angle periodically depends on *d*/*L* and is always close to $\theta \left( 0 \right) = 38.57\;{^{\circ}}$ (compare with Fig. 3n) as a consequence of the slight modification of the free cavity modes produced by the presence of the ML-TMD. In Fig. 4c, we plot the pump intensity threshold ${\it{I}}_{3Th}^{\left( i \right)}$ of the POs shown in Fig. 4b as a function of *d*/*L*. The marked periodic dependence of the intensity threshold on the location of the ML-TMD is particularly evident, together with the existence of minima and very large maxima. Such features can be easily understood by noting that at different locations inside the cavity, the ML-TMD experiences a spatially periodic cavity modal field (which is observed, as detailed above, to be slightly dependent on the location of the ML-TMD) and therefore shows minima and maxima for the intensity threshold at the anti-node and node positions (where the modal field strength is maximal and zero, respectively).

It is also worth stressing that such features are strictly a consequence of the two-dimensional character of the ML-TMD, which can additionally be exploited to tune and control the parametric oscillator behavior.

The novel PO utilizing ML-TMDs as nonlinear media are PM free because of the atomic size of the ML-TMDs. Several examples of POs with MoS_2_ can also be designed using other families of ML-TMDs, leading to qualitatively similar results. In the Supplementary Material, we compare the calculated dependence of the pump intensity threshold as a function of wavelength *λ*_3_ for parametric oscillators built using MoS_2_, WS_2_, and MoSe_2_, WSe_2_; we find that the chosen material affects the minimal threshold intensity in a given spectral range. One can optimize the choice of the material for a desired spectral content and threshold level. In this respect, we emphasize that these functionalities are enabled by the inherently large nonlinear surface conductivities of ML-TMDs. A heuristic comparison with standard photonic media may be accomplished by introducing an effective second-order nonlinear mixing susceptibility $\chi _{\mathrm {eff}}^{\left( 2 \right)}\left( {\omega _1,\omega _2} \right)$ for the ML-TMDs, which is found to be of the order $\chi _{\mathrm {eff}}^{\left( 2 \right)}\left( {\omega _1,\omega _2} \right) \approx 10^{ - 10}\;{\mathrm {mV}}^{ - 1}$ (≈2 orders of magnitude higher than that of LiNbO_3_, which is one of the most widespread and efficient materials used for second-order nonlinear optical functionalities^42^). Therefore, by using standard photonic media instead of ML-TMDs (in the envisaged micro-cavity), parametric oscillations would require a pump threshold that is at least 4 orders of magnitude higher (the threshold intensity depends inversely on the product $\left| {\tilde \sigma _{23}\tilde \sigma _{13}^ \ast } \right|$, see Materials and methods), and second-order nonlinear effects due to other photonic components of the proposed device are expected to be irrelevant.

A further degree of freedom offered by ML-TMDs lies in the electrical tunability afforded by the application of an external gate voltage, as depicted in Fig. 1c. The gate voltage increases the Fermi level and hence affects the nonlinearity and absorption because of electron–electron collisions in the conduction band (see Fig. 2d, f). Although electrical tunability of MX_2_ has not been hitherto experimentally demonstrated, to the best of our knowledge, we emphasize that such an additional degree of freedom is absent in traditional parametric oscillators. In the Supplementary Material, we calculate the pump intensity threshold as a function of the Fermi level of MoS_2_, and we show that the threshold may increase by one order of magnitude. Consequently, an external gate voltage can be used to switch-off PO at a fixed optical pump intensity, with potential for realization of rapid electrical modulation of the output signal and idler fields.

Finally, we emphasize that experimental realization of the discussed micron-sized phase-matching-free parametric oscillators is heavily facilitated by the inherent flexibility offered by these devices. Indeed, in contrast to traditional parametric oscillators, the key tunability (by means of the external pump incidence angle) unlocks the cavity size, which remains arbitrary. While the narrow angular selectivity found in our calculations can be easily overcome by using focused pump beams with finite size, the reflectivity of the Bragg mirrors heavily affects the parametric oscillation threshold. Thus, high-reflectivity Bragg mirrors with leakage ≈10^−4^ are desirable for reaching thresholds on the order of GW cm^−2^, which are achievable using pulsed infrared lasers with picosecond-like single pulse duration. Accurate control of the TMD layer number remains the only experimentally critical limiting factor: since TMDs with even layer numbers are centrosymmetric, it is imperative for the oscillator design to embed TMDs with an odd layer number. In addition, increasing the layer number hampers relaxation of the phase-matching condition; therefore, TMD monolayers are considered to be the best materials in terms of design optimization.

## Conclusions

POs can be excited in micron-sized cavities embedding ML-TMDs as nonlinear media at conventional pump intensities in a PM-free regime. The cavity design remains inherently free of the complexity imposed by the need for PM and can be used to realize doubly resonant PDC of signal and idler waves. The flexibility offered by such novel oscillator design enables the engineering of selective degenerate or non-degenerate down-converted excitations by simple modification of the incident angle of the pump field. Furthermore, electrical tunability of ML-TMDs can enable one to rapidly modulate the output signal and idler waves by shifting POs below the threshold. Based on our calculations, we demonstrate that novel parametric oscillators embedding ML-TMDs highlight a new technology for all applications in which highly miniaturized tunable sources are relevant, including environmental detection, security, biophysics, imaging and spectroscopy. PM-free ML-TMD microresonators can also be potentially used to realize micrometric sources of entangled photons when pumped slightly below the threshold, thus paving the way for the development of integrated quantum processors.

## Electronic supplementary material

SUPPLEMENTARY INFORMATION for Phase-matching-free parametric oscillators based on two-dimensional semiconductors
