# Supplementary material for: Phase-matching-free parametric oscillators based on two-dimensional semiconductors
Source: Light Sci Appl. 2018 May 18;7:5. doi: 10.1038/s41377-018-0011-3 (PMC6107017; doi:10.1038/s41377-018-0011-3)
Supplement: Supplementary file 1 — SUPPLEMENTARY INFORMATION for Phase-matching-free parametric oscillators based on two-dimensional semiconductors [file 41377_2018_11_MOESM1_ESM.pdf]

SUPPLEMENTARY INFORMATION for
Phase-matching-free parametric oscillators based on two dimensional semiconductors

Alessandro Ciattoni[1],* Andrea Marini[2,3,4],† Carlo Rizza[1,5], and Claudio Conti[3,6]
[1]*Consiglio Nazionale delle Ricerche, CNR-SPIN, Via Vetoio 10, 67100 L'Aquila, Italy*
[2]*Department of Physical and Chemical Sciences, University of L'Aquila, Via Vetoio 10, 67100 L'Aquila, Italy*
[3]*Institute for Complex Systems (ISC-CNR), Via dei Taurini 19, 00185, Rome, Italy*
[4]*ICFO-Institut de Ciencies Fotoniques, The Barcelona Institute of Science and Technology, 08860 Castelldefels (Barcelona), Spain*
[5]*Department of Industrial and Information Engineering and Economics, Via G. Gronchi 18, University of L'Aquila, I-67100 L'Aquila, Italy and*
[6]*Department of Physics, University Sapienza, Piazzale Aldo Moro 5, 00185, Rome, Italy*

Here we provide additional information on technical aspects of the theoretical methods used to model parametric down-conversion and the resulting phase-matching free resonant oscillations within the micro-cavities described in the main paper.

I. PARAMETRIC DOWN-CONVERSION OF MX_2

We calculate the linear and parametric down-conversion (PDC) mixing surface conductivities of monolayer (ML) transition metal dichalcogenides (TMDs) MX_2 (M = Mo, S, and X = S, Se) starting from a tight-binding (TB) description of the electronic band structure of these materials [1] and studying the light-driven electron dynamics by means of Bloch equations for the valence and conduction bands. Note that our approach is based on the independent-electron approximation and is fully justified at the frequencies considered in the main paper since they are far from exciton resonances happening at photon energies of $\simeq 1.5$ eV or higher [2, 3]. In addition, for the infrared photon energies considered, the relevant valence and conduction band regions affecting the infrared response are the ones closer to the band gap around the K and K' band edges, for which the full TB Hamiltonian can be approximated by a two-band $\mathbf{k}\cdot\mathbf{p}$ Hamiltonian $\mathcal{H}_0(\mathbf{k},\tau,s) = \mathcal{H}_1(\mathbf{k},\tau,s) + \mathcal{H}_2(\mathbf{k},\tau)$ [1], where

$$\mathcal{H}_1(\mathbf{k},\tau,s) = \begin{bmatrix} \Delta/2 & t_0 a(\tau k_x - i k_y) \\ t_0 a(\tau k_x + i k_y) & \tau s\Lambda - \Delta/2 \end{bmatrix}, \tag{1}$$

$$\mathcal{H}_2(\mathbf{k},\tau,s) = \begin{bmatrix} \gamma_1 a^2 k^2 & \gamma_3 a^2(\tau k_x + i k_y)^2 \\ \gamma_3 a^2(\tau k_x - i k_y)^2 & \gamma_2 a^2 k^2 \end{bmatrix}, \tag{2}$$

$$\tag{3}$$

and $\tau, s = \pm 1$ label non-degenerate valleys and spins, while $\mathbf{k} = (k_x \ k_y)$ indicates the electron wave-vector. The physical parameters of $\mathcal{H}_0(\mathbf{k},\tau,s)$ are obtained by fitting the $\mathbf{k}\cdot\mathbf{p}$ valence and conduction energy bands with the ones obtained from first-principles GW simulations [1] accounting for both non-degenerate valleys and spin-orbit coupling, and are listed in Table S1. We calculate the linear and PDC mixing conductivities of ML-TMDs by introducing

TABLE S1: Fitted constants of the the two-band $\mathbf{k}\cdot\mathbf{p}$ Hamiltonian $\mathcal{H}_0(\mathbf{k},\tau,s)$.

Constants	MoS_2	$MoSe_2$	WS_2	WSe_2
a (Å)	3.190	3.326	3.191	3.325
Δ (eV)	1.658	1.429	1.806	1.541
t_0 (eV)	0.933	0.768	1.196	1.016
Λ (eV)	0.073	0.091	0.211	0.228
γ_1 (eV)	0.351	0.291	0.443	0.404
γ_2 (eV)	−0.198	−0.191	−0.211	−0.216
γ_3 (eV)	−0.143	−0.099	−0.199	−0.150

*Electronic address: alessandro.ciattoni@spin.cnr.it
†Electronic address: andrea.marini@icfo.es

the time-dependent Hamiltonian $\mathcal{H}_0(\boldsymbol{\kappa}(t),\tau,s)$, where we have replaced the electron wave-vector with the minimum coupling prescription for the electron quasi-momentum $\hbar\boldsymbol{\kappa}(t)=\hbar\mathbf{k}+e\mathbf{A}(t)$, where $-e$ is the electron charge and $\mathbf{A}(t)$ is the electromagnetic potential vector accounting for pump, signal, and idler waves. With this prescription, we define unperturbed and interacting Hamiltonians $\mathcal{H}_0(\mathbf{k},\tau,s)$ and $\mathcal{H}_\mathrm{I}(\mathbf{k},\tau,s,t)$, respectively, and write the total Hamiltonian as $\mathcal{H}_\mathrm{T}(\mathbf{k},\tau,s,t)=\mathcal{H}_0[\boldsymbol{\kappa}(t),\tau,s]=\mathcal{H}_0(\mathbf{k},\tau,s)+\mathcal{H}_\mathrm{I}(\mathbf{k},\tau,s,t)$, where

$$\mathcal{H}_\mathrm{I}(\mathbf{k},\tau,s,t) \;=\; \frac{e}{\hbar}[D_xA_x(t)+D_yA_y(t)]+\frac{e^2}{\hbar^2}[D_{xx}A_x^2(t)+D_{xy}A_x(t)A_y(t)+D_{yy}A_y^2(t)], \tag{4}$$

and the interaction operators are explicitly given by

$$\begin{aligned}
D_x &= t_0a\tau\left[|\psi_\mathrm{V}\rangle\langle\psi_\mathrm{C}|+|\psi_\mathrm{C}\rangle\langle\psi_\mathrm{V}|\right],\\
D_y &= it_0a\left[|\psi_\mathrm{V}\rangle\langle\psi_\mathrm{C}|-|\psi_\mathrm{C}\rangle\langle\psi_\mathrm{V}|\right],\\
D_{xx} &= \gamma_1a^2|\psi_\mathrm{C}\rangle\langle\psi_\mathrm{C}|+\gamma_2a^2|\psi_\mathrm{V}\rangle\langle\psi_\mathrm{V}|+\gamma_3a^2\left[|\psi_\mathrm{V}\rangle\langle\psi_\mathrm{C}|+|\psi_\mathrm{C}\rangle\langle\psi_\mathrm{V}|\right],\\
D_{yy} &= \gamma_1a^2|\psi_\mathrm{C}\rangle\langle\psi_\mathrm{C}|+\gamma_2a^2|\psi_\mathrm{V}\rangle\langle\psi_\mathrm{V}|-\gamma_3a^2\left[|\psi_\mathrm{V}\rangle\langle\psi_\mathrm{C}|+|\psi_\mathrm{C}\rangle\langle\psi_\mathrm{V}|\right],\\
D_{xy} &= 2i\gamma_3a^2\tau\left[|\psi_\mathrm{C}\rangle\langle\psi_\mathrm{V}|-|\psi_\mathrm{V}\rangle\langle\psi_\mathrm{C}|\right].
\end{aligned} \tag{5}$$

In the expressions above we use the Dirac notation for the conduction $|\psi_\mathrm{C}\rangle$ and valence $|\psi_\mathrm{V}\rangle$ band eigenstates, and we approximate the matrix elements by their values at the band edges ($\mathbf{k}=0$). Inserting the Ansatz $|\psi\rangle=c_-|\psi_\mathrm{V}\rangle+c_+|\psi_\mathrm{C}\rangle$ in the time-dependent Schrödinger equation $i\hbar\partial_t|\psi\rangle=\mathcal{H}_\mathrm{T}|\psi\rangle$, and defining the inversion population $n_\mathbf{k}=|c_+|^2-|c_-|^2$ and the interband coherence $\rho_\mathbf{k}=c_+c_-^*$, one gets

$$\begin{aligned}
\dot\rho_\mathbf{k} \;&=\; -\frac{i}{\hbar}(E_\mathrm{C}-E_\mathrm{V})\rho_\mathbf{k}-\gamma\rho_\mathbf{k}+\frac{ie}{\hbar^2}n_\mathbf{k}\left\{D_x^\mathrm{CV}A_x(t)+D_y^\mathrm{CV}A_y(t)+\frac{e}{\hbar}[D_{xx}^\mathrm{CV}A_x^2(t)+D_{xy}^\mathrm{CV}A_x(t)A_y(t)+D_{yy}^\mathrm{CV}A_y^2(t)]\right\}+\\
&\quad+\frac{ie^2}{\hbar^3}\left[(D_{xx}^\mathrm{VV}-D_{xx}^\mathrm{CC})A_x^2(t)+(D_{yy}^\mathrm{VV}-D_{yy}^\mathrm{CC})A_y^2(t)\right]\rho_\mathbf{k},\\
\dot n_\mathbf{k} \;&=\; -\frac{4e}{\hbar^2}\mathrm{Im}\left\{\rho_\mathbf{k}\left[D_x^\mathrm{VC}A_x(t)+D_y^\mathrm{VC}A_y(t)+\frac{e}{\hbar}[D_{xx}^\mathrm{VC}A_x^2(t)+D_{xy}^\mathrm{VC}A_x(t)A_y(t)+D_{yy}^\mathrm{VC}A_y^2(t)]\right]\right\},
\end{aligned} \tag{6}$$

where $E_\mathrm{C}(\mathbf{k})$ and $E_\mathrm{V}(\mathbf{k})$ are the conduction and valence energy bands of the unperturbed Hamiltonian $\mathcal{H}_0$, $D_j^\mathrm{CV}=\langle\psi_\mathrm{C}|D_j|\psi_\mathrm{V}\rangle$ are the interaction matrix elements, and we have introduced a phenomenological relaxation rate $\gamma=10$ ps^{-1} accounting for coherence dephasing [4]. In order to obtain the PDC surface conductivities, we consider a coherent superposition of three monochromatic fields $\mathbf{E}(t)=\mathrm{Re}\left\{\mathbf{E}_1\mathrm{e}^{-i\omega_1t}+\mathbf{E}_2\mathrm{e}^{-i\omega_2t}+\mathbf{E}_3\mathrm{e}^{-i\omega_3t}\right\}$ with amplitudes $\mathbf{E}_1$, $\mathbf{E}_2$, and $\mathbf{E}_3$, and with angular frequencies ω_1, ω_2, and ω_3, respectively. In our notation, the field $\mathbf{E}_3$ indicates the external pump field, while $\mathbf{E}_1$, $\mathbf{E}_2$ label the down-converted signal and idler fields, respectively. The down-converted angular frequencies ω_1 and ω_2 are not independent, but are such that $\omega_1+\omega_2=\omega_3$ owing to energy conservation. The electromagnetic potential vector related to the coherent superposition of pump, signal, and idler waves is thus given by

$$\mathbf{A}(t)=\mathrm{Re}\left\{(\mathbf{E}_1/i\omega_1)\mathrm{e}^{-i\omega_1t}+(\mathbf{E}_2/i\omega_2)\mathrm{e}^{-i\omega_2t}+(\mathbf{E}_3/i\omega_3)\mathrm{e}^{-i\omega_3t}\right\}. \tag{7}$$

We then solve perturbatively the equations above in the vanishing temperature $T\to0$ and weak excitation limits such that $n_\mathbf{k}\approx-\Theta\left[E_\mathrm{C}(\mathbf{k})-E_\mathrm{F}\right]$, where $\Theta(x)$ indicates the Heaviside step function and E_F is the Fermi energy. Taking the Ansatz $\rho_\mathbf{k}=\sum_{j=\pm1,\pm2,\pm3}\rho_{|j|}^{(j/|j|)}\mathrm{e}^{i(j/|j|)\omega_{|j|}t}$ and disregarding generation of higher harmonics we obtain analytical expressions for the coefficients $\rho_{|j|}^{(j/|j|)}$, finding that the macroscopic surface current density given by

$$\begin{aligned}
\mathbf{K}(t) \;&=\; -\frac{e}{4\pi^2\hbar}\sum_{\tau,s=-1,1}\int_{-\infty}^{+\infty}dk_x\int_{-\infty}^{+\infty}dk_y\left[\langle\psi(t)|\nabla_\mathbf{k}\mathcal{H}_\mathrm{T}(t)|\psi(t)\rangle-\langle\psi_\mathrm{V}|\nabla_\mathbf{k}\mathcal{H}_\mathrm{T}(t)|\psi_\mathrm{V}\rangle\right]= \qquad (8)\\
&=\; -\frac{e}{2\pi^2\hbar}\sum_{\tau,s=-1,1}\int_{-\infty}^{+\infty}dk_x\int_{-\infty}^{+\infty}dk_y\mathrm{Re}\left\{\rho_\mathbf{k}(t)\left[\nabla_\mathbf{k}\mathcal{H}_0^\mathrm{VC}+\nabla_\mathbf{k}D_x^\mathrm{VC}\frac{e}{\hbar}A_x(t)+\nabla_\mathbf{k}D_y^\mathrm{VC}\frac{e}{\hbar}A_y(t)+\right.\right.\\
&\quad\left.+\nabla_\mathbf{k}D_{xx}^\mathrm{VC}\frac{e^2}{\hbar^2}A_x^2(t)+\nabla_\mathbf{k}D_{xy}^\mathrm{VC}\frac{e^2}{\hbar^2}A_x(t)A_y(t)+\nabla_\mathbf{k}D_{yy}^\mathrm{VC}\frac{e^2}{\hbar^2}A_y^2(t)\right]+\\
&\quad\left.+\Theta\left[E_\mathrm{F}-E_\mathrm{C}(\mathbf{k})\right]\left[D_{xx}^\mathrm{CC}\frac{e}{\hbar}A_x(t)+D_{yy}^\mathrm{CC}\frac{e}{\hbar}A_y(t)\right]\right\},
\end{aligned}$$

can be recast into

$$\mathbf{K}(t)=\mathrm{Re}\left\{\sum_{j=1}^{3}\left[\hat{\sigma}^{\mathrm{L}}(\omega_j)\mathbf{E}_j\mathrm{e}^{-i\omega_j t}\right]+\hat{\sigma}^{(1,2)}\mathbf{E}_1\mathbf{E}_2\mathrm{e}^{-i\omega_3 t}+\hat{\sigma}^{(1,3)}\mathbf{E}_1^*\mathbf{E}_3\mathrm{e}^{-i\omega_2 t}+\hat{\sigma}^{(2,3)}\mathbf{E}_2^*\mathbf{E}_3\mathrm{e}^{-i\omega_1 t}\right\}, \tag{9}$$

where $\hat{\sigma}^{\mathrm{L}}(\omega_j)$ $(j=1,2,3)$ and $\hat{\sigma}^{(l,m)}$ $(l,m=1,2,3)$ are the linear and PDC surface conductivity tensors, respectively, and we have neglected again generation of higher harmonics. Since centrosymmetry is broken along the y-direction in the notation used, the relevant components of the surface conductivity tensors for PDC are the ones such that pump, signal, and idler fields are polarized along the y-direction, for which

$$\sigma^{\mathrm{L}}_{yy}(\omega)=\frac{ie^2D^{\mathrm{CC}}_{yy}}{2\pi^2\hbar^2(\omega+i\gamma)}\sum_{\tau,s=\pm1}\int_{-\infty}^{+\infty}dk_x\int_{-\infty}^{+\infty}dk_y\Theta\left[E_{\mathrm{F}}-E_{\mathrm{C}}(\mathbf{k})\right]+ \tag{10}$$
$$+\frac{e^2}{4i\pi^2\hbar^2\omega}\sum_{\tau,s=\pm1}\int_{-\infty}^{+\infty}dk_x\int_{-\infty}^{+\infty}dk_y\Theta\left[E_{\mathrm{C}}(\mathbf{k})-E_{\mathrm{F}}\right]\left\{\frac{|D^{\mathrm{CV}}_y|^2}{[(E_{\mathrm{C}}-E_{\mathrm{V}})-\hbar(\omega+i\gamma)]}+\frac{|D^{\mathrm{CV}}_y|^2}{[(E_{\mathrm{C}}-E_{\mathrm{V}})+\hbar(\omega+i\gamma)]}\right\},$$
$$\sigma^{(1,2)}_{yyy}(\omega_1,\omega_2,\omega_3)=\frac{-e^3}{4\pi^2\hbar^3\omega_1\omega_2}\sum_{\tau,s=\pm1}\sum_{j=1}^{3}\int_{-\infty}^{+\infty}dk_x\int_{-\infty}^{+\infty}dk_y\Theta\left[E_{\mathrm{C}}(\mathbf{k})-E_{\mathrm{F}}\right]\left\{\frac{D^{\mathrm{CV}}_yD^{\mathrm{VC}}_{yy}}{[(E_{\mathrm{C}}-E_{\mathrm{V}})-\hbar(\omega_j+i\gamma)]}+\right.$$
$$\left.+\frac{D^{\mathrm{VC}}_yD^{\mathrm{CV}}_{yy}}{[(E_{\mathrm{C}}-E_{\mathrm{V}})+\hbar(\omega_j+i\gamma)]}\right\},$$
$$\sigma^{(1,3)}_{yyy}(\omega_1,\omega_2,\omega_3)=\frac{e^3}{4\pi^2\hbar^3\omega_1\omega_3}\sum_{\tau,s=\pm1}\int_{-\infty}^{+\infty}dk_x\int_{-\infty}^{+\infty}dk_y\Theta\left[E_{\mathrm{C}}(\mathbf{k})-E_{\mathrm{F}}\right]\left\{\frac{D^{\mathrm{CV}}_yD^{\mathrm{VC}}_{yy}}{[(E_{\mathrm{C}}-E_{\mathrm{V}})+\hbar(\omega_1-i\gamma)]}+\right. \tag{11}$$
$$+\frac{D^{\mathrm{VC}}_yD^{\mathrm{CV}}_{yy}}{[(E_{\mathrm{C}}-E_{\mathrm{V}})-\hbar(\omega_1-i\gamma)]}+\frac{D^{\mathrm{CV}}_yD^{\mathrm{VC}}_{yy}}{[(E_{\mathrm{C}}-E_{\mathrm{V}})+\hbar(\omega_2+i\gamma)]}+\frac{D^{\mathrm{VC}}_yD^{\mathrm{CV}}_{yy}}{[(E_{\mathrm{C}}-E_{\mathrm{V}})-\hbar(\omega_2+i\gamma)]}+$$
$$\left.+\frac{D^{\mathrm{CV}}_yD^{\mathrm{VC}}_{yy}}{[(E_{\mathrm{C}}-E_{\mathrm{V}})-\hbar(\omega_3+i\gamma)]}+\frac{D^{\mathrm{VC}}_yD^{\mathrm{CV}}_{yy}}{[(E_{\mathrm{C}}-E_{\mathrm{V}})+\hbar(\omega_3+i\gamma)]}\right\},$$
$$\sigma^{(2,3)}_{yyy}(\omega_1,\omega_2,\omega_3)=\frac{e^3}{4\pi^2\hbar^3\omega_2\omega_3}\sum_{\tau,s=\pm1}\int_{-\infty}^{+\infty}dk_x\int_{-\infty}^{+\infty}dk_y\Theta\left[E_{\mathrm{C}}(\mathbf{k})-E_{\mathrm{F}}\right]\left\{\frac{D^{\mathrm{CV}}_yD^{\mathrm{VC}}_{yy}}{[(E_{\mathrm{C}}-E_{\mathrm{V}})+\hbar(\omega_1+i\gamma)]}+\right. \tag{12}$$
$$+\frac{D^{\mathrm{VC}}_yD^{\mathrm{CV}}_{yy}}{[(E_{\mathrm{C}}-E_{\mathrm{V}})-\hbar(\omega_1+i\gamma)]}+\frac{D^{\mathrm{CV}}_yD^{\mathrm{VC}}_{yy}}{[(E_{\mathrm{C}}-E_{\mathrm{V}})+\hbar(\omega_2-i\gamma)]}+\frac{D^{\mathrm{VC}}_yD^{\mathrm{CV}}_{yy}}{[(E_{\mathrm{C}}-E_{\mathrm{V}})-\hbar(\omega_2-i\gamma)]}+\frac{D^{\mathrm{CV}}_yD^{\mathrm{VC}}_{yy}}{[(E_{\mathrm{C}}-E_{\mathrm{V}})-\hbar(\omega_3+i\gamma)]}+$$
$$\left.+\frac{D^{\mathrm{VC}}_yD^{\mathrm{CV}}_{yy}}{[(E_{\mathrm{C}}-E_{\mathrm{V}})+\hbar(\omega_3+i\gamma)]}\right\}.$$

Data reported in the main paper are obtained through the expressions above. In what follows, for convenience we will assume the simplified notation $\sigma_n=\sigma^{\mathrm{L}}_{yy}(\omega_n)$ and $\sigma_{nm}=\sigma^{(n,m)}_{yyy}(\omega_1,\omega_2,\omega_3)$ since the pump, signal, and idler electric fields are polarized in the y-direction for maximizing PDC within the micro-cavity.

II. EQUATIONS FOR THE OUTPUT FIELDS

In Fig.S1 we sketch the geometry of the parametric oscillator (PO) considered in our calculations. A dielectric (PMMA) slab of thickness L with a MX_2 monolayer lying on its left side (at $z=0$) is placed between two Bragg mirrors of thickness d (for convenience we choose the right mirror to be the reflected $z\to-z$ copy of the left one). The left side of the cavity is illuminated with an incident (i) pump field which is a monochromatic Transverse Electric (TE) plane wave of frequency ω_3 with incidence angle θ. In addition to the reflected (r) and transmitted (t) pump fields, due to PDC, the cavity also produces (r) and (t) TE plane waves at the frequencies ω_1 (signal) and ω_2 (idler) such that $\omega_3=\omega_1+\omega_2$. It is convenient to set

$$\begin{aligned}\omega_1&=\frac{1}{2}\left(\omega_3+\Delta\omega\right),\\ \omega_2&=\frac{1}{2}\left(\omega_3-\Delta\omega\right),\end{aligned} \tag{13}$$

since the note-beat frequency $\Delta\omega = \omega_1 - \omega_2$ is sufficient to label the signal and idler frequencies produced by a pump field of frequency ω_3. Conservation of transverse momentum of the three fields implies that the their complex amplitudes ($\sim e^{-i\omega_n t}$, $n = 1, 2, 3$) are

$$\begin{aligned} \mathbf{E}_n &= e^{i\frac{\omega_n}{c}x\sin\theta}\left[A_{ny}(z)\,\hat{\mathbf{e}}_y\right], \\ \mathbf{H}_n &= e^{i\frac{\omega_n}{c}x\sin\theta}\sqrt{\frac{\varepsilon_0}{\mu_0}}\left[A_{nx}(z)\,\hat{\mathbf{e}}_x + A_{ny}(z)\sin\theta\hat{\mathbf{e}}_z\right]. \end{aligned} \tag{14}$$

Accordingly the three two-component column vectors $(A_{nx}(z)\ A_{ny}(z))^T$ fully describe the field and in vacuum, i.e. outside the cavity, they are

$$\begin{cases} \begin{pmatrix} A_{nx} \\ A_{ny} \end{pmatrix} = E_n^{(i)}\begin{pmatrix} -\cos\theta \\ 1 \end{pmatrix} e^{i\frac{\omega_n}{c}(z+d)\cos\theta} + E_n^{(r)}\begin{pmatrix} \cos\theta \\ 1 \end{pmatrix} e^{-i\frac{\omega_n}{c}(z+d)\cos\theta}, & z < -d, \\ \begin{pmatrix} A_{nx} \\ A_{ny} \end{pmatrix} = E_n^{(t)}\begin{pmatrix} -\cos\theta \\ 1 \end{pmatrix} e^{i\frac{\omega_n}{c}(z-L-d)\cos\theta}, & z > L+d, \end{cases} \tag{15}$$

where $E_n^{(i)}$, $E_n^{(r)}$ and $E_n^{(t)}$ are field amplitudes with $E_1^{(i)} = E_2^{(i)} = 0$. Resorting to the transfer matrix approach, the fields at the left ($z = 0^-$) and right ($z = 0^+$) sides of the MX_2 monolayer are

$$\begin{aligned} \begin{pmatrix} A_{nx} \\ A_{ny} \end{pmatrix}_{z=0^-} &= F_n \begin{pmatrix} A_{nx} \\ A_{ny} \end{pmatrix}_{z=-d}, \\ \begin{pmatrix} A_{nx} \\ A_{ny} \end{pmatrix}_{z=0^+} &= B_n B'_n \begin{pmatrix} A_{nx} \\ A_{ny} \end{pmatrix}_{z=L+d}, \end{aligned} \tag{16}$$

where F_n, B_n and B'_n are the transfer matrix describing the forward, backward and backward propagations through the left Bragg mirror, the dielectric slab and the right Bragg mirror, respectively. The transfer matrix F_n of the left Bragg mirror is the (ordered) product of the transfer matrices of the slabs composing the mirror. For later convenience it is useful to represent this matrix as [5]

$$F_n = \begin{pmatrix} \frac{cq_n}{\omega_n\cos\theta}\left[\frac{t_n^{(L)}t_n^{(R)} + \left(1+r_n^{(L)}\right)\left(1-r_n^{(R)}\right)}{2t_n^{(R)}}\right] & \frac{cq_n}{\omega_n}\left[\frac{-t_n^{(L)}t_n^{(R)} + \left(1-r_n^{(L)}\right)\left(1-r_n^{(R)}\right)}{2t_n^{(R)}}\right] \\ \frac{1}{\cos\theta}\left[\frac{-t_n^{(R)}t_n^{(L)} + \left(1+r_n^{(L)}\right)\left(1+r_n^{(R)}\right)}{2t_n^{(R)}}\right] & \left[\frac{t_n^{(L)}t_n^{(R)} + \left(1-r_n^{(R)}\right)\left(1+r_n^{(R)}\right)}{2t_n^{(R)}}\right] \end{pmatrix}, \tag{17}$$

where $q_n = \frac{\omega_n}{c}\sqrt{\varepsilon(\omega_n) - \sin^2\theta}$ are the longitudinal wavenumbers inside the dielectric slab, $\epsilon(\omega)$ is the relative permittivity of the dielectric slab whereas $r_n^{(L)}, t_n^{(L)}, r_n^{(R)}, t_n^{(R)}$ are the complex reflectivities r and transmittivities t for

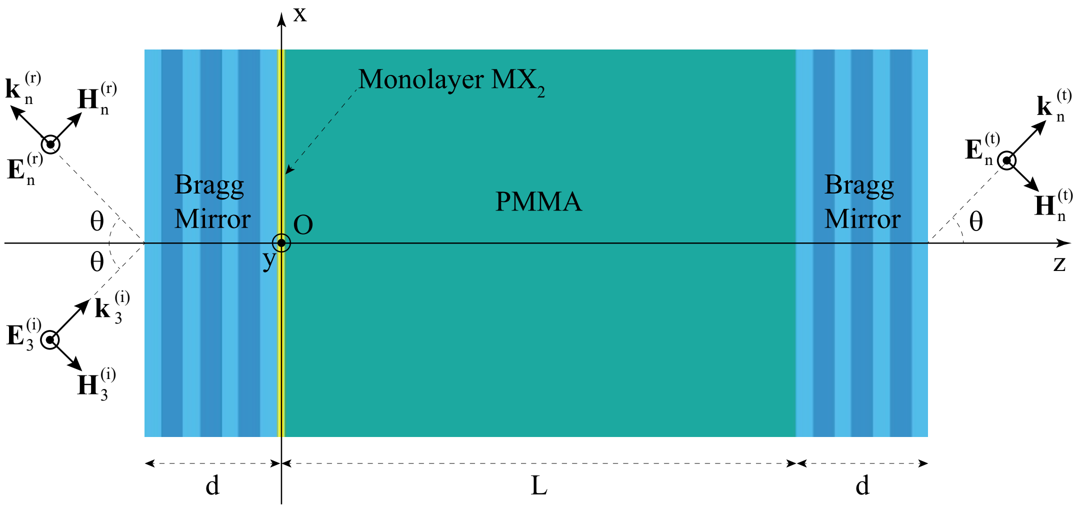

FIG. S1: Parametric oscillator geometry.

left (L) and right (R) illumination of the left Bragg mirror (with vacuum and the dielectric on its left and right sides, respectively). The other relevant transfer matrices are [5]

$$\begin{aligned} B_n &= \begin{pmatrix} \cos(q_n L) & i\frac{cq_n}{\omega_n}\sin(q_n L) \\ i\frac{\omega_n}{cq_n}\sin(q_n L) & \cos(q_n L) \end{pmatrix}, \\ B'_n &= \begin{pmatrix} 1 & 0 \\ 0 & -1 \end{pmatrix} F_n \begin{pmatrix} 1 & 0 \\ 0 & -1 \end{pmatrix}, \end{aligned} \tag{18}$$

where the last of Eqs. (18) is a consequence of the fact that the right Bragg mirror is the reflected image of the left one. Using Eqs. (15), Eqs. (16) yield

$$\begin{aligned} \begin{pmatrix} A_{nx} \\ A_{ny} \end{pmatrix}_{z=0^-} &= \begin{pmatrix} V_{nx}^{(i)} \\ V_{ny}^{(i)} \end{pmatrix} E_n^{(i)} + \begin{pmatrix} V_{nx}^{(r)} \\ V_{ny}^{(r)} \end{pmatrix} E_n^{(r)}, \\ \begin{pmatrix} A_{nx} \\ A_{ny} \end{pmatrix}_{z=0^+} &= \begin{pmatrix} V_{nx}^{(t)} \\ V_{ny}^{(t)} \end{pmatrix} E_n^{(t)}, \end{aligned} \tag{19}$$

where

$$\begin{aligned} \begin{pmatrix} V_{nx}^{(i)} \\ V_{ny}^{(i)} \end{pmatrix} &= \frac{1}{t_n^{(R)}} \begin{pmatrix} \frac{cq_n}{\omega_n}\left[-t_n^{(L)}t_n^{(R)} + r_n^{(L)}\left(r_n^{(R)} - 1\right)\right] \\ t_n^{(L)}t_n^{(R)} - r_n^{(L)}\left(r_n^{(R)} + 1\right) \end{pmatrix}, \\ \begin{pmatrix} V_{nx}^{(r)} \\ V_{ny}^{(r)} \end{pmatrix} &= \frac{1}{t_n^{(R)}} \begin{pmatrix} -\frac{cq_n}{\omega_n}\left(r_n^{(R)} - 1\right) \\ r_n^{(R)} + 1 \end{pmatrix}, \\ \begin{pmatrix} V_{nx}^{(t)} \\ V_{ny}^{(t)} \end{pmatrix} &= \frac{1}{t_n^{(R)}} \begin{pmatrix} \frac{cq_n}{\omega_n}\left(r_n^{(R)}e^{iq_nL} - e^{-iq_nL}\right) \\ \left(r_n^{(R)}e^{iq_nL} + e^{-iq_nL}\right) \end{pmatrix}. \end{aligned} \tag{20}$$

The monolayer of MX_2 in the presence of the above TE electromagnetic field hosts a surface current whose harmonic complex amplitudes are $\mathbf{K}_n = K_n\hat{\mathbf{e}}_y$ where

$$\begin{aligned} K_1 &= \left[\sigma_1 E_{1y} + \sigma_{23}E_{2y}^* E_{3y}\right]_{z=0}, \\ K_2 &= \left[\sigma_2 E_{2y} + \sigma_{13}E_{1y}^* E_{3y}\right]_{z=0}, \\ K_3 &= \left[\sigma_3 E_{3y} + \sigma_{12}E_{1y} E_{2y}\right]_{z=0}, \end{aligned} \tag{21}$$

showing both a linear and a quadratic response to the electric field. The effect of such surface current on the field is provided by the electromagnetic boundary conditions at $z = 0$, namely $\hat{\mathbf{e}}_z \times \{[\mathbf{E}_n]_{z=0^+} - [\mathbf{E}_n]_{z=0^-}\} = \mathbf{0}$ and $\hat{\mathbf{e}}_z \times \{[\mathbf{H}_n]_{z=0^+} - [\mathbf{H}_n]_{z=0^-}\} = \mathbf{K}_n$ which, using Eqs. (14), can be casted within the two-component column vector description as

$$\begin{aligned} \begin{pmatrix} A_{1x} \\ A_{1y} \end{pmatrix}_{z=0^+} - \begin{pmatrix} A_{1x} \\ A_{1y} \end{pmatrix}_{z=0^-} &= \begin{pmatrix} \tilde{\sigma}_1 A_{1y} + \tilde{\sigma}_{23}A_{2y}^* A_{3y} \\ 0 \end{pmatrix}_{z=0^+}, \\ \begin{pmatrix} A_{2x} \\ A_{2y} \end{pmatrix}_{z=0^+} - \begin{pmatrix} A_{2x} \\ A_{2y} \end{pmatrix}_{z=0^-} &= \begin{pmatrix} \tilde{\sigma}_2 A_{2y} + \tilde{\sigma}_{13}A_{1y}^* A_{3y} \\ 0 \end{pmatrix}_{z=0^+}, \\ \begin{pmatrix} A_{3x} \\ A_{3y} \end{pmatrix}_{z=0^+} - \begin{pmatrix} A_{3x} \\ A_{3y} \end{pmatrix}_{z=0^-} &= \begin{pmatrix} \tilde{\sigma}_3 A_{3y} + \tilde{\sigma}_{12}A_{1y} A_{2y} \\ 0 \end{pmatrix}_{z=0^+}, \end{aligned} \tag{22}$$

where, for each component, we have set $\tilde{\sigma} = \sqrt{\mu_0/\varepsilon_0}\sigma$, where ε_0 and μ_0 indicate the dielectric permittivity and magnetic permeability of vacuum, respectively. After inserting Eqs. (19) along with $E_1^{(i)} = E_2^{(i)} = 0$ into Eqs. (22)

we obtain

$$
\begin{aligned}
V_{1x}^{(t)}E_1^{(t)} - V_{1x}^{(r)}E_1^{(r)} &= \tilde{\sigma}_1 V_{1y}^{(t)}E_1^{(t)} + \tilde{\sigma}_{23} V_{2y}^{(t)*} V_{3y}^{(t)} E_2^{(t)*} E_3^{(t)}, \\
V_{1y}^{(t)}E_1^{(t)} - V_{1y}^{(r)}E_1^{(r)} &= 0, \\
V_{2x}^{(t)}E_2^{(t)} - V_{2x}^{(r)}E_2^{(r)} &= \tilde{\sigma}_2 V_{2y}^{(t)}E_2^{(t)} + \tilde{\sigma}_{13} V_{1y}^{(t)*} V_{3y}^{(t)} E_1^{(t)*} E_3^{(t)}, \\
V_{2y}^{(t)}E_2^{(t)} - V_{2y}^{(r)}E_2^{(r)} &= 0, \\
V_{3x}^{(t)}E_3^{(t)} - V_{3x}^{(i)}E_3^{(i)} - V_{3x}^{(r)}E_3^{(r)} &= \tilde{\sigma}_3 V_{3y}^{(t)}E_3^{(t)} + \tilde{\sigma}_{12} V_{1y}^{(t)} V_{2y}^{(t)} E_1^{(t)} E_2^{(t)}, \\
V_{3y}^{(t)}E_3^{(t)} - V_{3y}^{(i)}E_3^{(i)} - V_{3y}^{(r)}E_3^{(r)} &= 0,
\end{aligned} \tag{23}
$$

which are six equations for the six unknown amplitudes $E_n^{(t)}$, $E_n^{(r)}$ of the signal, idler and pump fields transmitted and reflected by the cavity illuminated by incident pump field of amplitude $E_3^{(i)}$. The second, fourth and sixth of Eqs. (23) can be written as

$$
\begin{aligned}
E_1^{(r)} &= \frac{V_{1y}^{(t)}}{V_{1y}^{(r)}} E_1^{(t)}, \\
E_2^{(r)} &= \frac{V_{2y}^{(t)}}{V_{2y}^{(r)}} E_2^{(t)}, \\
E_3^{(r)} &= \frac{V_{3y}^{(t)}}{V_{3y}^{(r)}} E_3^{(t)} - \frac{V_{3y}^{(i)}}{V_{3y}^{(r)}} E_3^{(i)},
\end{aligned} \tag{24}
$$

showing that the reflected fields can be evaluated once the transmitted fields are known. Substituting the reflected fields from Eqs. (24) into Eqs. (23) and using Eqs. (20) we eventually get

$$
\begin{aligned}
\Delta_1 Q_1 + \tilde{\sigma}_{23} Q_2^* Q_3 &= 0, \\
\Delta_2 Q_2 + \tilde{\sigma}_{13} Q_1^* Q_3 &= 0, \\
\Delta_3 Q_3 + \tilde{\sigma}_{12} Q_1 Q_2 &= P_3,
\end{aligned} \tag{25}
$$

where

$$
\begin{aligned}
Q_n &= \left(\frac{r_n^{(R)} e^{iq_n L} + e^{-iq_n L}}{t_n^{(R)}} \right) E_n^{(t)}, \\
P_3 &= \left(\frac{2 t_3^{(R)} \cos\theta}{r_3^{(R)} + 1} \right) E_3^{(i)}, \\
\Delta_n &= \tilde{\sigma}_n - \frac{c q_n}{\omega_n} \left(\frac{r_n^{(R)} - 1}{r_n^{(R)} + 1} + \frac{r_n^{(R)} e^{iq_n L} - e^{-iq_n L}}{r_n^{(R)} e^{iq_n L} + e^{-iq_n L}} \right).
\end{aligned} \tag{26}
$$

Equations (25) are the basic equations for the output fields. Note that Eqs. (25) have been derived without resorting to any electromagnetic approximation commonly used in cavity nonlinear optics (e.g. slowly varying amplitude approximation, decoupled approximation for counter-propagating waves, etc.) and this is a consequence of the fact the overall nonlinear response of the MX_2 monolayer is confined to a single plane.

III. DOUBLY RESONANT PARAMETRIC OSCILLATION CONDITIONS

Equations (25) provide the amplitudes Q_n (proportional to the amplitudes of the transmitted signal, idler and pump fields) for a given amplitude P_3 (proportional to the amplitude of the incident pump field). Note that they always admit the solution

$$
Q_1 = Q_2 = 0, \quad Q_3 = \frac{P_3}{\Delta_3}, \tag{27}
$$

which describes the linear response of the cavity to the pump field without parametric oscillations (POs) in turn characterized by $Q_1 \neq 0$ and $Q_2 \neq 0$. On the other hand, the first and the complex conjugate of the second of Eqs. (25) are a linear system for Q_1 and Q_2^* and it admits nontrivial solutions only if its determinant vanishes (see Section IV below) or

$$|Q_3|^2 = \frac{\Delta_1 \Delta_2^*}{\tilde{\sigma}_{23}\tilde{\sigma}_{13}^*}. \tag{28}$$

This condition entails the occurrence of POs since if it is fulfilled, Eqs. (25) have solutions with $Q_1 \neq 0$ and $Q_2 \neq 0$ which are stable whereas the linear one ($Q_1 = Q_2 = 0$) becomes unstable. Since the right hand side of Eq. (28) is generally a complex number, it is evident that POs can occur *only if* such complex number is real and positive or

$$\frac{\Delta_1 \Delta_2^*}{\tilde{\sigma}_{23}\tilde{\sigma}_{13}^*} = \left| \frac{\Delta_1 \Delta_2^*}{\tilde{\sigma}_{23}\tilde{\sigma}_{13}^*} \right|, \tag{29}$$

which, due to Eqs. (13) and (26), is a constraint joining the cavity length L, the note-beat frequency $\Delta\omega$, and the incident angle θ. Geometrically, Eq. (29) represents a surface Σ of the three-dimensional cavity state space $(L, \Delta\omega, \theta)$, and its typical slices ($\theta = 0$ or $L = L_0$) are illustrated in Figs. 3a, 3j and 3l of the main paper (green curves). At each point of the surface Σ, PO ignites if $|Q_3|$ is sufficiently large to fulfill Eq. (28). Therefore, considering an experiment where the incident pump $|P_3|$ is gradually increased starting from the linear regime where $Q_1 = Q_2 = 0$, the PO threshold is obtained by inserting the linear solution $Q_3 = \frac{P_3}{\Delta_3}$ into Eq. (28), thus obtaining

$$\left(|P_3|^2\right)_{th} = \frac{\Delta_1 \Delta_2^*}{\tilde{\sigma}_{23}\tilde{\sigma}_{13}^*} |\Delta_3|^2, \tag{30}$$

which, through the second of Eqs. (26) and the relation $I_3^{(i)} = \frac{1}{2}\sqrt{\frac{\epsilon_0}{\mu 0}} \left| E_3^{(i)} \right|^2$, entails the intensity threshold for the incident pump. Note that the denominator of the right hand side of Eq. (30) contains the nonlinear conductivities $\tilde{\sigma}_{23}$ and $\tilde{\sigma}_{13}$ whose moduli are so small to generally yield exceedingly large and unfeasible intensity thresholds. A viable way for observing POs thus necessitates the identification of the points $(L, \Delta\omega, \theta)$ of the surface Σ for which $|\Delta_1|$ and $|\Delta_2|$ are very close to zero. An inspection of the third of Eqs. (26) reveals that $|\Delta_n|$ can not be small if $\left| r_n^{(R)} \right|$ is not close to 1. Therefore, by choosing Bragg mirrors with high reflectivities, the third of Eqs. (26) can be expanded up to the first order in the parameter $1 - \left| r_n^{(R)} \right| \ll 1$ thus getting

$$\Delta_n = \left[\tilde{\sigma}_n - i \left(\frac{2cq_n}{\omega_n} \right) \frac{\sin \Gamma_n}{\cos \Gamma_n + \cos(q_n L)} \right] + \left\{ \left(\frac{2cq_n}{\omega_n} \right) \frac{1 + (\cos \Gamma_n + 2i \sin \Gamma_n) \cos(q_n L)}{[\cos \Gamma_n + \cos(q_n L)]^2} \right\} \left(1 - \left| r_n^{(R)} \right| \right), \tag{31}$$

where $\Gamma_n = q_n L + \arg r_n^{(R)}$. The minima of $|\Delta_n|$ are easily seen to occur for $\Gamma_n = m\pi$ (where m is any integer) which is exactly the cavity resonance condition for the frequency ω_n [5] and where, up to the first order of $1 - \left| r_n^{(R)} \right|$,

$$\Delta_n = \tilde{\sigma}_n + \left[\left(\frac{2cq_n}{\omega_n} \right) \frac{1}{1 + \cos\left(\arg r_n^{(R)}\right)} \right] \left(1 - \left| r_n^{(R)} \right| \right). \tag{32}$$

Therefore, as for standard POs based on bulk nonlinear media, the pump intensity threshold is here minimum when one or more of the three fields meet the cavity resonant condition. Designing a Bragg mirror with high reflectivity for both signal and idler fields is relatively simple (see the Section III below) and therefore in this paper we consider only doubly resonant (DR) states where the signal resonance (SR) and idler resonance (IR) conditions

$$\begin{aligned} q_1 L + \arg r_1^{(R)} &= m_1 \pi, \\ q_2 L + \arg r_2^{(R)} &= m_2 \pi, \end{aligned} \tag{33}$$

are both achieved whereas the pump is non-resonant. Such two equations represents two surfaces Σ_1 and Σ_2 of the space $(L, \Delta\omega, \theta)$ whose typical slices are reported in Figs. 3a, 3j and 3l of the main text (black and red lines respectively) and whose intersection describes the DR cavity states where $|\Delta_1 \Delta_2|$ is minimum. The (nontrivial) intersection among the three surfaces Σ, Σ_1 and Σ_2 is the set of the DRPO cavity states with feasible intensity

threshold. Note that if Σ_1 and Σ_2 intersect at a specific $(L, 0, \theta)$ point this point also belongs to the surface Σ since for $\Delta\omega = 0$ Eqs. (29) is trivially satisfied since evidently $\omega_1 = \omega_2$, $\Delta_1 = \Delta_2$ and $\sigma_{23} = \sigma_{13}$. In other words a degenerate ($\omega_1 = \omega_2$) DR state always supports a PO which we refer to as a degenerate DRPO. In addition, note that if $|\text{Re}\,\tilde{\sigma}_n| \ll |\text{Im}\,\tilde{\sigma}_n|$ and $|\text{Re}\,\tilde{\sigma}_{nm}| \ll |\text{Im}\,\tilde{\sigma}_{nm}|$, if both Eqs. (33) are satisfied with $\Delta\omega \neq 0$, Eq. (32) implies that $\left|\text{Im}\left(\frac{\Delta_1\Delta_2^*}{\tilde{\sigma}_{23}\tilde{\sigma}_{13}^*}\right)\right| \ll \left|\text{Re}\left(\frac{\Delta_1\Delta_2^*}{\tilde{\sigma}_{23}\tilde{\sigma}_{13}^*}\right)\right|$ so that, remarkably, if both linear and nonlinear absorption are small the non-degenerate DR states are always very close to PO states. As a consequence the non-degenerate DRPOs with feasible intensity threshold are associated to those points of the surface Σ which are as close as possible to points of the intersection between the surface Σ_1 and Σ_2. Both degenerate and non-degenerate DRPO states are labelled with a dashed disk in Figs. 3a, 3j and 3l of the main text.

IV. BRAGG MIRROR DESIGN

The Bragg mirror is a periodic structure composed of N bi-layers whose dielectric materials have refractive indexes $n^{(a)}$ and $n^{(b)}$ and thicknesses a and b. If the layers' thicknesses are chosen to satisfy the Bragg interference condition

$$an^{(a)} = bn^{(b)} = \frac{\pi c}{2\bar{\omega}}, \tag{34}$$

the mirror has (for normal incidence $\theta = 0$), a spectral stop-band centered at $\bar{\omega}$ whose width is proportional to the refractive index contrast $|n^{(a)} - n^{(b)}|$ [5]. Within the stop-band the mirror reflectivity is very large, the larger N the closer $|r^{(R)}(\omega)|$ to 1. As explained above in Section II, in order for one of the three fields (pump, signal and idler) to be resonant, it is necessary a very large reflectivity of the Bragg mirror at the field angular frequency, or in other words the frequency ω_n has to lie within the mirror stop-band. As noted above, the degenerate DR states with $\Delta\omega = 0$ (where, from Eqs. (13), $\omega_1 = \omega_2 = \omega_3/2$) rigorously supports POs so that it is convenient to set the center of the mirror stop-bad at $\bar{\omega} = \omega_3/2$. Due to the refractive index contrast $|n^{(a)} - n^{(b)}|$, this condition assures that both signal and idler fields experience very large mirror reflectivity in a range of $\Delta\omega$ and can accordingly be resonant at the same time. On the other hand the pump frequency ω_3 is twice the central mirror frequency $\bar{\omega}$ and requiring also the pump to resonate would require very large refractive contrast. To avoid this difficulty we have chosen to leave the pump out of resonance.

In the analysis reported in Fig.3 of the main text, we have set as pump wavelength $\lambda_3 = 780$ nm. For the Bragg Mirror we have chosen the refractive indexes $n_a = 1.2$ and $n_b = 2.5$ so that, in order to have the center of the stop-band at $\bar{\omega} = \omega_3/2$ we have chosen the thicknesses $a = \lambda_3/(2n^{(a)}) = 325$ nm and $b = \lambda_3/(2n^{(b)}) = 156$ nm. We have also set $N = 8$ for dealing with an efficient, feasible and compact Bragg mirror of length $d = N(a+b) = 3848$ nm. Using the transfer matrix approach, the complex reflectivity $r^{(R)}$ (for normal incidence $\theta = 0$) of the Bragg mirror which has vacuum and the dielectric at its left and right sides, respectively, is easily evaluated and we plot its absolute value and argument in panel (a) and (b), respectively, of Fig.S2. Accordingly, the Bragg mirror stop-band is centered at $\omega_3/2$ and its spectral width is $\simeq 0.22\,\omega_3$. As a consequence, if ω_1 and ω_2 lie within this stop-band, signal and idler waves can resonate simultaneously since $r_1^{(B)} = r^{(B)}(\omega_1)$ and $r_2^{(B)} = r^{(B)}(\omega_2)$ have moduli very close to 1. The mirror stop-band width therefore yields the note-beat frequency range $0 \leq \Delta\omega < 0.22\omega$, which is the one considered in the analysis reported in Fig.3 of the main text. Note that ω_3 lies outside the mirror stop-band and thus the pump field does not resonate.

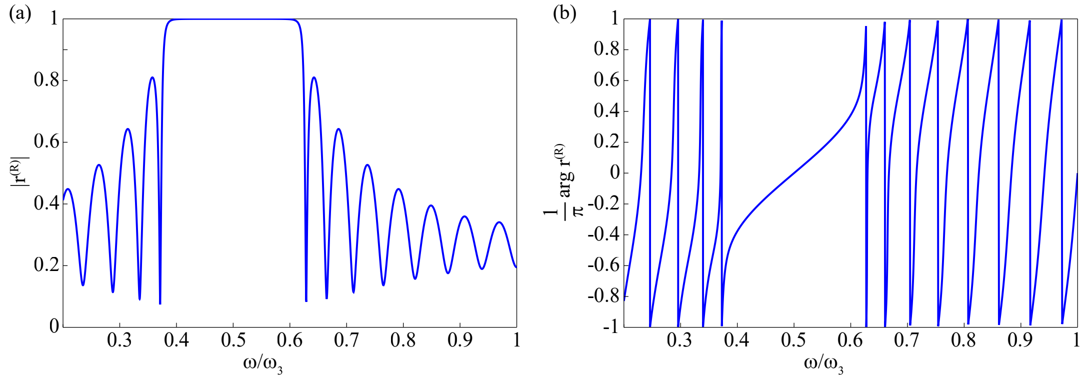

FIG. S2: Absolute value (a) and argument (b) of the Bragg Mirror complex reflectivity $r^{(R)}$ for normal incidence $\theta = 0$.

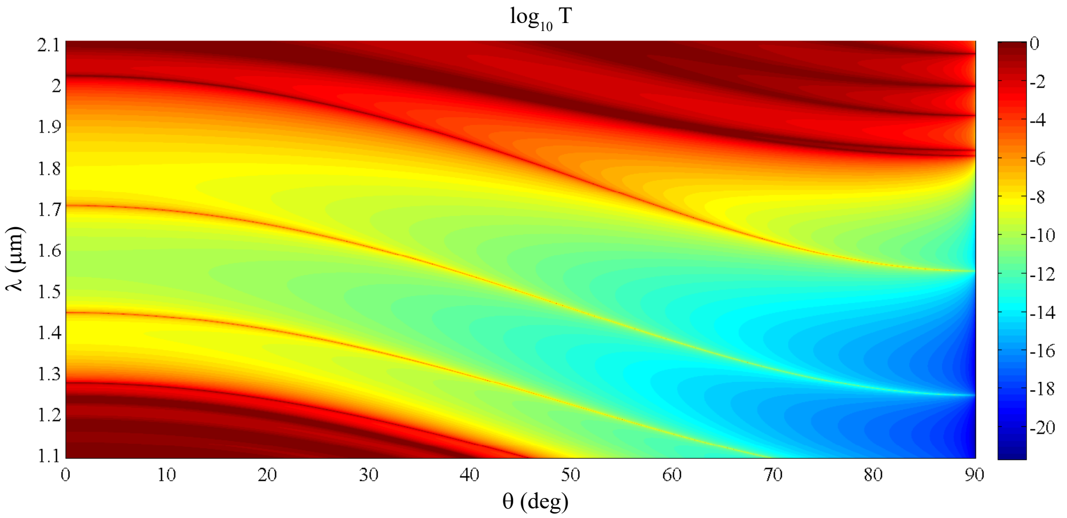

FIG. S3: Log plot of the linear cavity transmissity T as a function of the incidence angle θ and the wavelength λ for a cavity of thickness $L = 3.05\lambda_3 = 2.379$ nm with MoS_2 as TMD monolayer.

V. LINEAR CAVITY TRANSMISSIVITY

In order to justify that the PO pump intensity threshold is minimum when one (or more) of the three fields meets the cavity resonant condition, it is worth discussing the linear transmission spectrum of the cavity. In the low-intensity regime the third of Eqs.(25) yields

$$Q = \frac{P}{\Delta} \tag{35}$$

where the subscript 3 has been dropped since we are here focusing on the linear cavity behavior. Using Eqs.(26), the cavity linear transmissivity is easily obtained and it is given by

$$T = \left| \frac{E^{(t)}}{E^{(i)}} \right|^2 = \frac{4 \left(t^{(R)}\right)^4 \cos^2\theta}{\left| 2\frac{cq}{\omega} \left[\left(r^{(R)}\right)^2 e^{iqL} - e^{-iqL} \right] - \tilde{\sigma} \left(r^{(R)} + 1 \right) \left[r^{(R)} e^{iqL} + e^{-iqL} \right] \right|^2},$$

which evidently is very similar to the familiar Fabry-Perot transmissivity with an additional contribution (in the denominator) arising from the surface conductivity $\tilde{\sigma}$ of the TMD monolayer. In Fig.S3 we plot T as a function of the incidence angle θ and the wavelength λ for a cavity of thickness $L = 3.05\lambda_3 = 2.379$ nm whose Bragg mirros are those discussed in the above section and with MoS_2 as TMD monolayer. Since the mirrors' stop band is centered at $\bar{\lambda} = 2\lambda_3 = 1560$ nm, it is evident that T is rather small for wavelengths close to $\bar{\lambda}$. On the other hand, cavity resonances where T is close to 1 occur around $\bar{\lambda}$, as expected. The fact that such resonances correspond to the minima of Δ in Eq.(35) supports the above identification of the minimum pump intensity threshold POs with the cavity resonances.

VI. OUTPUT FIELDS

In order to evaluate the PO output fields $E_n^{(t)}$, Eqs. (25) have to be solved for a given incident pump field $E_3^{(i)}$. POs are characterized by $Q_1 \neq 0$ and $Q_2 \neq 0$ for a given $Q_3 \neq 0$. First note that Eqs. (25) are left invariant by the gauge transformation

$$\begin{aligned} Q_1 &\to Q_1 e^{i\theta}, \\ Q_2 &\to Q_2 e^{-i\theta}, \end{aligned} \tag{36}$$

and this implies that for a given P_3 there are infinite pairs (Q_1, Q_2), all with the same $\Psi = \arg Q_1 + \arg Q_2$. In other words, the phase difference $\Phi = \arg Q_1 - \arg Q_2$ is not set by the pump field P_3. Evidently, such symmetry is

spontaneously broken in actual experiments where a single pair (Q_1, Q_2) (i.e. a single value of Φ) is selected by the specific way chosen to trigger POs.

In the case of POs, the first and the complex conjugate of the second of Eqs. (25) can be casted as

$$\begin{aligned} \frac{Q_1}{Q_2^*} &= -\frac{\tilde{\sigma}_{23} Q_3}{\Delta_1}, \\ \frac{Q_1}{Q_2^*} &= -\frac{\Delta_2^*}{\tilde{\sigma}_{13}^* Q_3^*}, \end{aligned} \tag{37}$$

whose consistency requires their right and left hand sides to coincide or

$$|Q_3|^2 = \frac{\Delta_1 \Delta_2^*}{\tilde{\sigma}_{23} \tilde{\sigma}_{13}^*} \tag{38}$$

which is the PO oscillation condition of Section II. [see Eq. (28)]. The right hand side of Eq. (38) is a positive real number if and only if the complex numbers $\Delta_1/\tilde{\sigma}_{23}$ and $\Delta_2/\tilde{\sigma}_{13}$ have the same argument φ or

$$\begin{aligned} \Delta_1 &= \tilde{\sigma}_{23} \left| \frac{\Delta_1}{\tilde{\sigma}_{23}} \right| e^{i\varphi}, \\ \Delta_2 &= \tilde{\sigma}_{13} \left| \frac{\Delta_2}{\tilde{\sigma}_{13}} \right| e^{i\varphi}, \end{aligned} \tag{39}$$

which are equivalent to Eq. (29) of Section II so that, considering only those states for which Eqs. (39) are satisfied, Eqs. (25) yield

$$\begin{aligned} \left| \frac{\Delta_1}{\tilde{\sigma}_{23}} \right| e^{i\varphi} Q_1 + Q_2^* Q_3 &= 0, \\ \left| \frac{\Delta_2}{\tilde{\sigma}_{13}} \right| e^{i\varphi} Q_2 + Q_1^* Q_3 &= 0, \\ \Delta_3 Q_3 + \tilde{\sigma}_{12} Q_1 Q_2 &= P_3. \end{aligned} \tag{40}$$

To solve this equation, after noting that Eqs. (37) require that $\left|\frac{Q_1}{Q_2}\right|^2 = \left|\frac{\Delta_2 \tilde{\sigma}_{23}}{\Delta_1 \tilde{\sigma}_{13}}\right|$ and exploiting the above discussed gauge symmetry, we set

$$\begin{aligned} Q_1 &= \sqrt{\left| \frac{\Delta_2}{\tilde{\sigma}_{13}} \right|} |Q| \, e^{i\frac{1}{2}(\Psi+\Phi)}, \\ Q_2 &= \sqrt{\left| \frac{\Delta_1}{\tilde{\sigma}_{23}} \right|} |Q| \, e^{i\frac{1}{2}(\Psi-\Phi)}, \end{aligned} \tag{41}$$

where we have used the symbol $|Q|$ to stress that this quantity is real. Inserting Eqs. (41) into Eqs. (40) yield

$$\begin{aligned} Q_3 &= -\sqrt{\left| \frac{\Delta_1 \Delta_2}{\tilde{\sigma}_{23} \tilde{\sigma}_{13}} \right|} e^{i(\Psi+\varphi)}, \\ |Q|^2 + \frac{\Delta_3}{\tilde{\sigma}_{12}} \sqrt{\left| \frac{\tilde{\sigma}_{23} \tilde{\sigma}_{13}}{\Delta_1 \Delta_2} \right|} e^{-i\Psi} Q_3 &= \sqrt{\left| \frac{\tilde{\sigma}_{23} \tilde{\sigma}_{13}}{\Delta_1 \Delta_2} \right|} \frac{P_3}{\tilde{\sigma}_{12}} e^{-i\Psi}. \end{aligned} \tag{42}$$

Note that the first two of Eqs. (40) both reduces to the first of Eqs. (42) through the change of variables given by Eqs. (41) and this is a consequence of the necessary Eq. (39). Substituting Q_3 from the first of Eqs. (42) into the second we get

$$|Q|^2 - \frac{\Delta_3}{\tilde{\sigma}_{12}} e^{i\varphi} = \sqrt{\left| \frac{\tilde{\sigma}_{23} \tilde{\sigma}_{13}}{\Delta_1 \Delta_2} \right|} \frac{P_3}{\tilde{\sigma}_{12}} e^{-i\Psi}, \tag{43}$$

which is a single complex equation for $|Q|$ and Ψ. After equating the square-moduli of the left and right sides of this equation we get

$$|Q|^4 - 2\mathrm{Re}\left(\frac{\Delta_3}{\tilde{\sigma}_{12}} e^{i\varphi} \right) |Q|^2 + \left(\left| \frac{\Delta_3}{\tilde{\sigma}_{12}} e^{i\varphi} \right|^2 - \left| \frac{\tilde{\sigma}_{23} \tilde{\sigma}_{13}}{\Delta_1 \Delta_2} \right| \left| \frac{P_3}{\tilde{\sigma}_{12}} \right|^2 \right) = 0, \tag{44}$$

which is a biquadratic equation for $|Q|$ whose solutions are

$$|Q| = \sqrt{\mathrm{Re}\left(\frac{\Delta_3}{\tilde{\sigma}_{12}}e^{i\varphi}\right) + \xi\sqrt{\left|\frac{\tilde{\sigma}_{23}\tilde{\sigma}_{13}}{\Delta_1\Delta_2}\right|\left|\frac{P_3}{\tilde{\sigma}_{12}}\right|^2 - \mathrm{Im}^2\left(\frac{\Delta_3}{\tilde{\sigma}_{12}}e^{i\varphi}\right)}}, \quad (45)$$

where $\xi = \pm 1$. Note that, due to the ξ factor, generally there are two $|Q|$ corresponding to a $|P_3|$ and hence bistable POs can in principle occur. In addition, it is fundamental stressing that $|Q|$ is real and hence Eq. (45) provides its value only if the arguments of the square roots are positive. Before discussing the range of $|P_3|$ where this is the case (see below), we assume $|Q|$ real and we deduce the output field amplitudes. Equation (43) yields

$$e^{i\Psi} = \sqrt{\left|\frac{\tilde{\sigma}_{23}\tilde{\sigma}_{13}}{\Delta_1\Delta_2}\right|}\frac{P_3}{\tilde{\sigma}_{12}\left(|Q|^2 - \frac{\Delta_3}{\tilde{\sigma}_{12}}e^{i\varphi}\right)}, \quad (46)$$

which is consistent since the modulus of its right hand side, due to Eq. (45), is equal to 1. Hence Eqs. (41) and the first of Eqs. (42) eventually yield

$$\begin{aligned}
Q_1 &= \zeta\left|\frac{\Delta_2\tilde{\sigma}_{23}}{\Delta_1\tilde{\sigma}_{13}}\right|^{1/4}\sqrt{\frac{P_3}{\tilde{\sigma}_{12}\left(|Q|^2 - \frac{\Delta_3}{\tilde{\sigma}_{12}}e^{i\varphi}\right)}}e^{i\frac{\Phi}{2}}|Q|, \\
Q_2 &= \zeta\left|\frac{\Delta_1\tilde{\sigma}_{13}}{\Delta_2\tilde{\sigma}_{23}}\right|^{1/4}\sqrt{\frac{P_3}{\tilde{\sigma}_{12}\left(|Q|^2 - \frac{\Delta_3}{\tilde{\sigma}_{12}}e^{i\varphi}\right)}}e^{-i\frac{\Phi}{2}}|Q|, \\
Q_3 &= \frac{P_3}{\tilde{\sigma}_{12}\left(|Q|^2 - \frac{\Delta_3}{\tilde{\sigma}_{12}}e^{i\varphi}\right)}e^{i(\varphi+\pi)}, \quad (47)
\end{aligned}$$

where $\zeta = \pm 1$ and the principal branch is assumed for all the complex square-roots.

Note that the output fields of Eqs. (47) satisfy Eq. (38) so that they describe all the possible cavity POs whenever they exist or, in other words, whenever $|Q|$ of Eq. (45) is a positive real number. Such requirement evidently sets a range for the input pump intensity $|P_3|^2$ and there are four different cases corresponding to the two values of ξ and of the two signs of $\mathrm{Re}\left(\frac{\Delta_3}{\tilde{\sigma}_{12}}e^{i\varphi}\right)$. The results of this analysis are reported in the following table.

	$\mathrm{Re}\left(\frac{\Delta_3}{\tilde{\sigma}_{12}}e^{i\varphi}\right) > 0$	$\mathrm{Re}\left(\frac{\Delta_3}{\tilde{\sigma}_{12}}e^{i\varphi}\right) < 0$
$\xi = 1$	$|P_3|^2 > \left(|P_3|^2\right)_-$	$|P_3|^2 > \left(|P_3|^2\right)_{th}$
$\xi = -1$	$\left(|P_3|^2\right)_- < |P_3|^2 < \left(|P_3|^2\right)_{th}$	no $|P_3|^2$

Here we have set

$$\left(|P_3|^2\right)_- = \left|\frac{\Delta_1\Delta_2}{\tilde{\sigma}_{23}\tilde{\sigma}_{13}}\right|\mathrm{Im}^2\left(\Delta_3\frac{|\tilde{\sigma}_{12}|}{\tilde{\sigma}_{12}}e^{i\varphi}\right) < \left|\frac{\Delta_1\Delta_2}{\tilde{\sigma}_{23}\tilde{\sigma}_{13}}\right||\Delta_3|^2 = \left(|P_3|^2\right)_{th}. \quad (48)$$

Therefore at each state where PO can occur (i.e. at a each point of the surface Σ) the scenario is the following one. If $\mathrm{Re}\left(\frac{\Delta_3}{\tilde{\sigma}_{12}}e^{i\varphi}\right) < 0$, there is only one PO (with $\xi = 1$) that effectively starts when Eq. (30) is satisfied, thus confirming the analysis of Section III. On the other hand, if $\mathrm{Re}\left(\frac{\Delta_3}{\tilde{\sigma}_{12}}e^{i\varphi}\right) > 0$ the scenario changes qualitatively since in this case there are two allowed POs (with $\xi = 1$ and $\xi = -1$) when $\left(|P_3|^2\right)_- < |P_3|^2 < \left(|P_3|^2\right)_{th}$ and a single PO (with $\xi = 1$) when Eq. (30) is satisfied. As a consequence in this case POs also exist *below* the threshold.

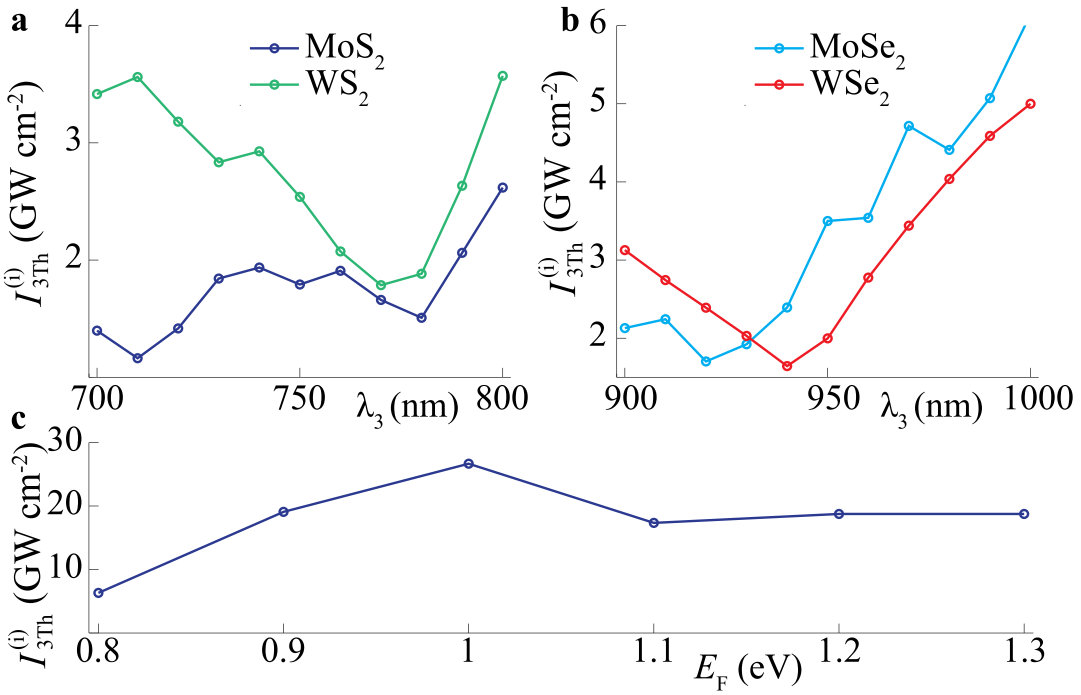

FIG. S4: Parametric Oscillation Thresholds. a,b Pump wavelength λ_3 dependence of the pump intensity thresholds for parametric oscillators embedding MoS_2, WS_2 (**a**) and $MoSe_2$, WSe_2 (b). c Pump intensity threshold of a parametric oscillator embedding MoS_2 as a function of the Fermi level. The thresholds are associated to non-degenerate ($\Delta\omega = 0$) POs and they have been calculated for cavities whose length L is equal to the pump wavelength λ_3.

In order to grasp the reason why the sub-threshold POs have not been entailed in Section III, note that in the case $\mathrm{Re}\left(\frac{\Delta_3}{\tilde{\sigma}_{12}} e^{i\varphi}\right) > 0$, if $|P_3|^2 = \left(|P_3|^2\right)_-$, Eq. (45) implies that $|Q| = \sqrt{\mathrm{Re}\left(\frac{\Delta_3}{\tilde{\sigma}_{12}} e^{i\varphi}\right)} \neq 0$ so that $Q_1 \neq 0$ and $Q_2 \neq 0$. In other words in this situation the signal and idler fields do not vanish *at the threshold* and accordingly this case is ruled out from the reasoning of Section III where the threshold has been obtained for PO oscillation starting from the linear regime. In a realistic experiment PO is switched on starting from the linear regime, with the intensity threshold given by Eq. (30). However, once PO ignites, by changing the pump intensity, incidence angle θ, or the cavity length, we argue that one can in principle access sub-threshold PO states. In every design considered in the main paper we have focused on the case $\mathrm{Re}\left(\frac{\Delta_3}{\tilde{\sigma}_{12}} e^{i\varphi}\right) < 0$, where sub-threshold PO does not occur.

VII. PUMP INTENSITY THRESHOLDS

In Fig. S4 we compare the calculated pump intensity thresholds versus the pump wavelength λ_3 for parametric oscillators embedding MoS_2, WS_2 (Fig. S4a) and $MoSe_2$, WSe_2 (Fig. S4b). Note that, while the minimal pump intensity threshold occurs at $\lambda_3 \approx 780$ nm for MoS_2 and WS_2, it shifts to $\lambda_3 \approx 940$ nm for $MoSe_2$ and WSe_2. None of the ML-TMDs examined enables feasible PO with low pump intensity threshold at optical frequencies owing to the enlarged absorption in this frequency range, which is the main responsible for oscillation quenching. In addition, at optical frequencies such materials exhibit exciton resonances [2] (not taken into account in our theoretical approach) that are also detrimental for POs owing to the enhanced absorption they are accompanied with. In Fig. S4c we plot the pump intensity threshold as a function of the Fermi level of MoS_2, showing that it can be increased efficiently. Thus, the external gate voltage quenches POs when the optical pump is fixed and fast modulation of the output signal and idler fields can be achieved with novel parametric oscillators embedding ML-TMDs.

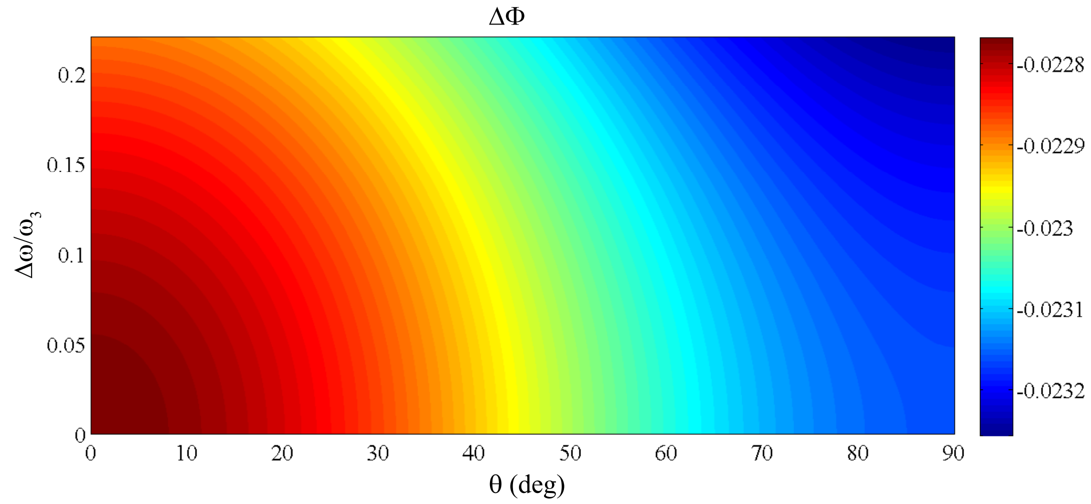

FIG. S5: Plot of the phase-mismatch among the pump, signal and idler waves $\Delta\Phi$ for a MoS_2 monolayer as a function of the incidence angle θ and the normalized detuning $\Delta\omega/\omega_3$ for $\omega_3 = 2\pi c/\lambda_3$ at $\lambda_3 = 780$ nm.

VIII. SURFACE CONDUCTIVITY PHASE DELAY AND RELAXATION OF THE PHASE-MATCHING CONSTRAINT

Up to now we have investigated the parametric oscillator behavior through a full electromagnetic analysis where the TMD monolayer has been modelled exploiting the well-established matching-conditions approach (see Eqs.(22)). As explained above, within such description the phase matching constrain simply does not show up since the absence of propagation through the TMD monolayer does not yield destructive interference among the three waves. However, even though very thin, the actual TMD monolayer has finite thickness and hence it is worth discussing the impact of the phase delay of the three fields resulting from the monolayer finite thickness on the relaxation of the phase-matching constraint we have discussed in the paper. To this end, we model the MoS_2 as an infinite dielectric layer of thinkness $d_M \simeq 0.65$ nm and dielectric permittivity

$$\varepsilon_M(\omega) = 1 + \frac{i\sigma(\omega)}{\omega\epsilon_0 d_M} \tag{49}$$

where $\sigma(\omega)$ is the above discussed monolayer surface conductivity. The longitudinal wave-vector within the layer is

$$k_M(\omega) = \frac{\omega}{c}\sqrt{\varepsilon_M(\omega) - \sin^2\theta} \tag{50}$$

so that the phase-mismatch among the three waves due to the finite monolayer thickness is

$$\Delta\Phi = \mathrm{Re}\left[k_M(\omega_3) - k_M(\omega_1) - k_M(\omega_2)\right] d_M. \tag{51}$$

In Fig.(S5) we plot $\Delta\Phi$ for a MoS_2 monolayer as a function of the incidence angle θ and the normalized detuning $\Delta\omega/\omega_3$ (see Eqs.(13)) for $\omega_3 = 2\pi c/\lambda_3$ at $\lambda_3 = 780$ nm. Note that $|\Delta\Phi|$ is *uniformly* much smaller than one hand hence it produces a tiny destructive interference among the three waves that we have actually neglected in the above treatment.

IX. PARAMETERS USED IN THE NUMERICAL SIMULATIONS

For completeness and clarity purposes we list here the values of all the parameters held fixed in the numerical simulations hitherto discussed.

Wavelengths	Pump Wavelength	$\lambda_3 = 780\,\text{nm}$
	Signal Wavelength Range	$1274\,\text{nm} < \lambda_1 < 1560\,\text{nm}$
	Idler Wavelength Range	$1560\,\text{nm} < \lambda_2 < 1996\,\text{nm}$
Bragg Mirrors	Layers' Thicknesses	$a = 325\,\text{nm}$, $b = 156\,\text{nm}$
	Layers' Refractive Indexes	$n_a = 1.2$, $n_b = 2.5$
	Number of Bilayers	$N = 8$
	Total Mirror Thickness	$d = 3848\,\text{nm}$
Dielectric Slab (PMMA)	Pump Dielectric Permittivity	$\varepsilon(\lambda_3) = 2.231$
	Signal Dielectric Permittivity	$\varepsilon(\lambda_1) = 2.220$
	Idler Dielectric Permittivity	$\varepsilon(\lambda_2) = 2.220$

The values of the surface linear and nonlinear conductivities of the MoS_2 monolayer are reported in Fig.2 of the main text.

[1] Liu GB, Shan WY, Yao Y, Yao W, Xiao D Three-band tight-binding model for monolayers of group-VIB transition metal dichalcogenides. *Phys Rev B* 2013; **88**: 085433.

[2] Ugeda MM, Bradley AJ, Shi SF, da Jornada FH, Zhang Y, Qiu DY, Ruan W, Mo SK, Hussain Z, Shen ZX, Wang F, Louie SG, Crommie MF Giant bandgap renormalization and excitonic effects in a monolayer transition metal dichalcogenide semiconductor. *Nat Materials* 2014; **13**: 1091-1095.

[3] Ceballos F, Cui Q, Bellusa MZ, Zhao H Exciton formation in monolayer transition metal dichalcogenides. *Nanoscale* 2016; **8**: 11681-11688.

[4] Yin X, Ye Z, Chenet DA, Ye Y, O'Brien K, Hone JC, Zhang X Edge Nonlinear Optics on a MoS2 Atomic Monolayer *Science* 2014; **344**: 488-490.

[5] Kavokin AV, Baumberg JJ, Malpuech G, Laussy FP *Microcavities* (Oxford University Press, USA 2008).
